# Insulin-Induced Recurrent Hypoglycemia Up-Regulates Glucose Metabolism in the Brain Cortex of Chemically Induced Diabetic Rats

**DOI:** 10.3390/ijms222413470

**Published:** 2021-12-15

**Authors:** Susana Cardoso, Paula I. Moreira

**Affiliations:** 1 CNC—Center for Neuroscience and Cell Biology, University of Coimbra, 3004-504 Coimbra, Portugal; susana.cardoso@cnc.uc.pt; 2 IIIU—Institute for Interdisciplinary Research, University of Coimbra, 3030-789 Coimbra, Portugal; 3 CIBB—Center for Innovative Biomedicine and Biotechnology, University of Coimbra, 3004-504 Coimbra, Portugal; 4 Institute of Physiology, Faculty of Medicine, University of Coimbra, 3000-370 Coimbra, Portugal

**Keywords:** brain cortex, chemically induced diabetes, recurrent hypoglycemia, mitochondria, glucose metabolism, signaling pathways

## Abstract

Diabetes is a chronic metabolic disease that seriously compromises human well-being. Various studies highlight the importance of maintaining a sufficient glucose supply to the brain and subsequently safeguarding cerebral glucose metabolism. The goal of the present work is to clarify and disclose the metabolic alterations induced by recurrent hypoglycemia in the context of long-term hyperglycemia to further comprehend the effects beyond brain harm. To this end, chemically induced diabetic rats underwent a protocol of repeatedly insulin-induced hypoglycemic episodes. The activity of key enzymes of glycolysis, the pentose phosphate pathway and the Krebs cycle was measured by spectrophotometry in extracts or isolated mitochondria from brain cortical tissue. Western blot analysis was used to determine the protein content of glucose and monocarboxylate transporters, players in the insulin signaling pathway and mitochondrial biogenesis and dynamics. We observed that recurrent hypoglycemia up-regulates the activity of mitochondrial hexokinase and Krebs cycle enzymes (namely, pyruvate dehydrogenase, alpha-ketoglutarate dehydrogenase and succinate dehydrogenase) and the protein levels of mitochondrial transcription factor A (TFAM). Both insults increased the nuclear factor erythroid 2–related factor 2 (NRF2) protein content and induced divergent effects in mitochondrial dynamics. Insulin-signaling downstream pathways were found to be down-regulated, and glycogen synthase kinase 3 beta (GSK3β) was found to be activated through both decreased phosphorylation at Ser9 and increased phosphorylation at Y216. Interestingly, no changes in the levels of cAMP response element-binding protein (CREB), which plays a key role in neuronal plasticity and memory, were caused by hypoglycemia and/or hyperglycemia. These findings provide experimental evidence that recurrent hypoglycemia, in the context of chronic hyperglycemia, has the capacity to evoke coordinated adaptive responses in the brain cortex that will ultimately contribute to sustaining brain cell health.

## 1. Introduction

Diabetes mellitus, a metabolic disease hallmarked by uncontrolled high blood glucose levels, has become an epidemic disease with an exponential increase all over the world [1]. According to the latest International Diabetes Federation Diabetes Atlas, there are approximately 463 million people in the world currently living with diabetes [2]. Among the main types of the disease, type 1 diabetes (T1D) is described to affect 5–10% of diagnosed cases, which is characterized by a widespread autoimmune destruction of insulin-secreting pancreatic β-cells. For this reason, this type of diabetes entails, since its diagnosis, a substitution therapy regimen consisting of multiple daily exogenous insulin injections, frequent blood glucose tests and careful dietary monitoring (e.g., carbohydrate counting) [3]. While this therapeutic plan is required for the survival of T1D individuals, the limitations of exogenous insulin therapy include the frequent occurrence of glycemic excursions and recurrent episodes of hypoglycemia, situations that, in due course, will contribute to the development and/or exacerbation of debilitating secondary complications, some of which are life-threatening [4].

It has long been recognized that the brain is a highly energy-demanding organ, consuming more than 20% of the total energy produced by the organism. In proportion to its size, it consumes more energy than any other tissue [5]. Due to this brain dependency almost exclusively on glucose, the occurrence of hypoglycemia and glycemic variations may be a threat for brain higher functions, such as cognitive performance [6,7,8]. Indeed, there is epidemiological evidence for links between diabetes and dementia [9,10]. For neuronal cells, this fuel requirement is mainly fulfilled by mitochondria, the multifunctional life-sustaining organelle that ensures the required energy supply through the production of adenosine triphosphate (ATP) via oxidative phosphorylation [11]. Within the brain, energy generated by mitochondria is used for the overall maintenance of cellular processes, the buffering of presynaptic calcium (Ca^2+^), neuronal growth, axonal branching and to ensure synaptic transmission [5]. Compelling data from us and others have provided evidence that diabetes and its glucose variation-mediated brain effects are strongly associated with disturbed mitochondrial homeostasis. As reported, different aspects of abnormal mitochondrial function (e.g., altered mitochondrial content and morphology, increased production of reactive oxygen species and altered redox system machinery, and altered energy metabolism) seem to exacerbate brain damage under peripheral variations in glucose levels [12,13,14,15,16,17].

Understanding the effects of dysglycemic conditions in the brain metabolism is of clinical and general importance as many diabetic patients experience daily glycemic fluctuations. Importantly, over time, many of these patients will develop a loss of counter-regulatory responses, becoming unable to recognize and promptly react to subsequent hypoglycemic episodes [18]. In this work, we intend to disclose the metabolic alterations induced by recurrent hypoglycemia occurring in the context of long-term hyperglycemia to further comprehend the effects beyond brain damage. For this purpose, experiments were carried out in chemically induced diabetic rats, which were subjected to repeated injections of insulin in order to induce recurrent bouts of hypoglycemia. Brain cortical tissue and isolated mitochondria were used to evaluate several steps of glucose metabolism and key mitochondrial homeostasis parameters. In brief, our data indicate that brain cortical tissue is differently impacted by different glycemic insults, which are the major effects observed in hypoglycemic rats. It was observed that recurrent hypoglycemia evokes significant alterations in the brain cortical metabolism, mitochondria content and dynamics, and significantly impacts insulin-mediated downstream signaling pathways. Ultimately, these (mal) adaptive responses will determine cells’ behavior and fate under these metabolic disturbances.

## 2. Results

### 2.1. Characterization of Experimental Groups

The diabetes phenotype was confirmed in chemically induced diabetic rats by significant elevations in blood glucose levels and glycated hemoglobin and a concomitant decrease in body weight when compared to age-matched control rats (Table 1). The insulin-induced hypoglycemic group showed a significant reduction in blood glucose levels, reflecting the values at the peak of hypoglycemic episodes (Table 1). Interestingly, in comparison with diabetic rats, insulin administration to induce recurrent episodes of hypoglycemia caused a significant recovery in body weight and a significant decrease in glycated hemoglobin values (Table 1). No significant alterations were detected in the brain weight of the experimental animals (Table 1).

### 2.2. Long-Term Hyperglycemia Down-Regulates GLUT3 Protein Levels in Brain Cortical Tissue

**Figure 1:**
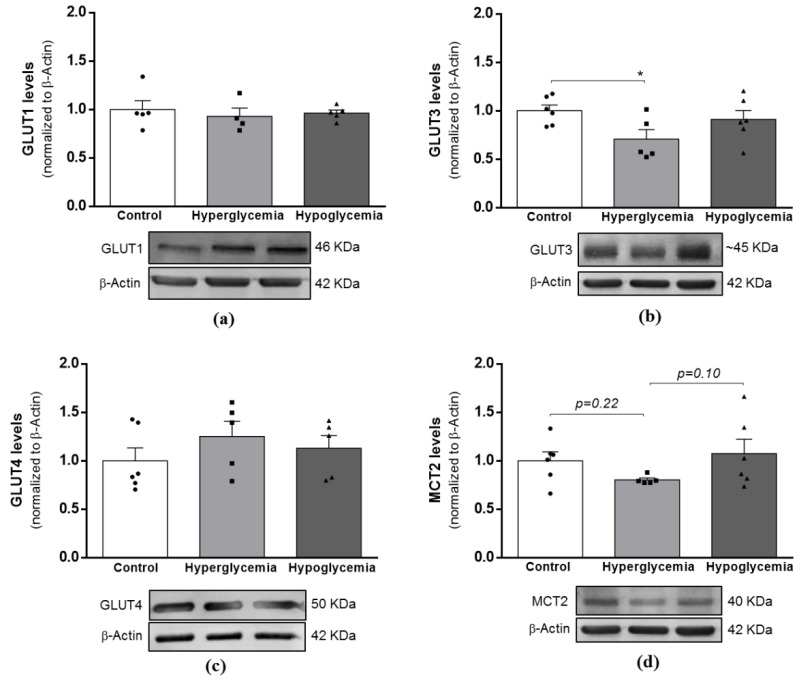
Effects of long-term hyperglycemia and insulin-induced recurrent hypoglycemia in the expression levels of brain glucose and monocarboxylate transporters. Western blot analyses of (**a**) GLUT1, (**b**) GLUT3, (**c**) GLUT4 and (**d**) MTC2 protein levels after normalization with β-Actin. Bars in graphs represent the mean ± SEM of the quantification of brain cortical samples from 4–6 rats under each condition. Representative immunoblots for β-actin are shown as loading control (for uncropped images of the Western blots, please see Supplementary Data). A * *p* < 0.05 was considered as a statistically significant difference. GLUT—Glucose transporter; MCT2—Monocarboxylate transporter 2.

The main energy substrate for the brain is glucose, which must be delivered from the systemic circulation and transported across the blood–brain barrier (BBB) to reach brain tissues through specialized glucose transporters (GLUTs) [19]. Under certain circumstances (e.g., hypoglycemia), the brain may also use alternative nonglucose substrates (e.g., lactate and pyruvate) to up-regulate the monocarboxylate transporters (MCT) [20]. Our densitometry quantification of the Western blots showed that neither hyperglycemia nor hypoglycemia significantly impacts the GLUT1 (Figure 1a), GLUT4 (Figure 1c) or MCT2 (Figure 1d) protein content. However, GLUT3 expression levels significantly decreased in the brain cortical tissue of hyperglycemic animals (Figure 1b). These observations suggest the occurrence of distinct responses to different peripheral alterations in glucose levels.

### 2.3. Recurrent Hypoglycemia Increases Mitochondrial Hexokinase Activity

**Figure 2:**
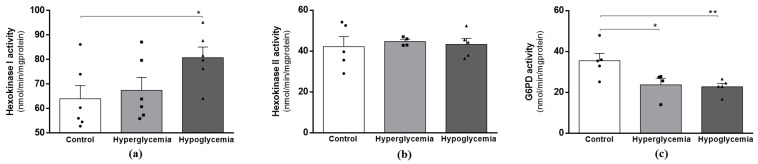
Effects of long-term hyperglycemia and insulin-induced recurrent hypoglycemia on the first steps of the metabolism of glucose. Hexokinase activity in (**a**) brain cortical isolated mitochondria and in (**b**) brain cortical tissue lysates from control, hyperglycemic and insulin-induced recurrent hypoglycemic rats. (**c**) G6PD activity in brain cortical tissue lysates from control, hyperglycemic and insulin-induced recurrent hypoglycemic rats. Data represent the mean ± SEM of the quantification of samples from 4–6 animals under each condition studied. ** *p* < 0.01 and * *p* < 0.05 were considered statistically significant differences. G6PD—Glucose-6-phosphate dehydrogenase.

After its uptake to (brain) cells, glucose is phosphorylated to glucose-6-phosphate by hexokinase (HK), the first committed step in glucose metabolism. HK has different isoforms with different subcellular distributions and metabolic roles; it can be attached to mitochondria (HKI), coupling cytosolic glycolysis to mitochondrial oxidative phosphorylation, or it can be in the cytoplasm (HKII), providing glucose-6-phosphate for the pentose phosphate pathway via glucose-6-phosphate dehydrogenase (G6PD) [21]. Herein, we observed that the cytoplasmic HKII activity remained unchanged in both hyperglycemic and hypoglycemic animals (Figure 2b), whereas the activity of mitochondrial HKI was significantly increased in the hypoglycemic animals (Figure 2a). We also observed a significant decrease in the activity of G6PD, the initiating step of the pentose phosphate pathway (PPP), under both dysglycemic conditions (Figure 2c). These observations suggest that under recurrent hypoglycemic conditions, glucose is preferentially channeled into the energy-producing catabolic process of glycolysis to increase and/or preserve the brain glucose metabolism.

### 2.4. Recurrent Hypoglycemia Increases Krebs Cycle Activity

**Figure 3:**
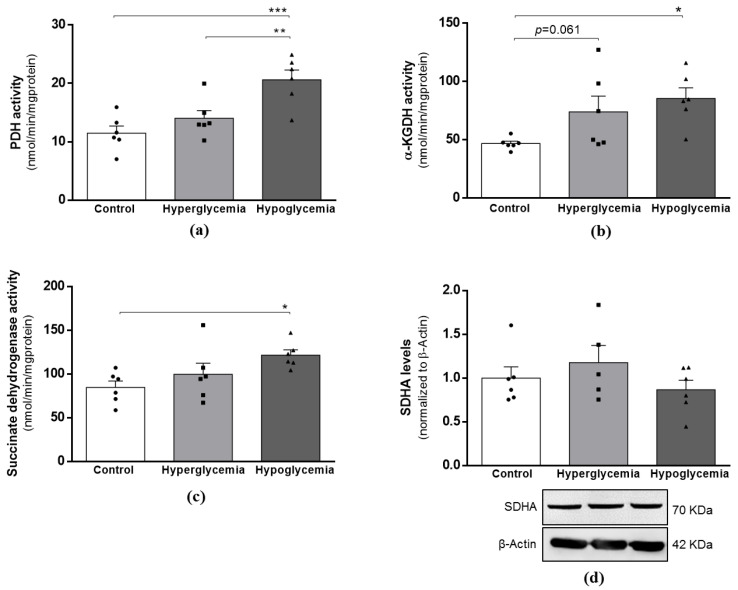
Effects of long-term hyperglycemia and insulin-induced recurrent hypoglycemia on the activity and expression of key Krebs cycle enzymes. (**a**) PDH activity, (**b**) α-KGDH activity and (**c**) SDH activity in brain cortical mitochondria from control, hyperglycemic and insulin-induced recurrent hypoglycemic rats. (**d**) SDH expression levels in brain cortical tissue extracts from control, hyperglycemic and insulin-induced recurrent hypoglycemic rats. Data represent the mean ± SEM of the quantification of brain cortical samples from 5–6 animals under each condition studied. When applied, representative immunoblots are shown below the graph, and β-Actin represents the loading control (for uncropped images of the Western blot, please see Supplementary Data). **** *p* < 0.001, ** *p* < 0.01 and * *p* < 0.05 were considered statistically significant differences. PDH—Pyruvate dehydrogenase; α-KGDH—Alpha-ketoglutarate dehydrogenase; SDH—Succinate dehydrogenase.

After observing significant alterations in the first steps of glucose metabolism, our next step was to examine the enzymatic activity of key proteins involved in the mitochondrial tricarboxylic acid (TCA) cycle. By spectrophotometry, we observed a significant increase in the enzymatic activity of pyruvate dehydrogenase (PDH; Figure 3a), alpha-ketoglutarate dehydrogenase (α-KGDH; Figure 3b) and of succinate dehydrogenase (SDH; Figure 3c) in brain cortical mitochondria from hypoglycemic animals. Furthermore, α-KGDH activity presented a trended increase in the hyperglycemic group (Figure 3b). Importantly, the increased activity of SDH was not due to alterations in the protein levels of the enzyme as the densitometric analysis remained unchanged in both experimental groups (Figure 3d). It is likely that the activity of citrate synthase, a classical mitochondrial marker, was not affected by any of the treatments (Supplementary Data), suggesting that the observed results are not due to the altered mitochondrial content. These data suggest that, in response to peripheral glucose levels alterations, all the metabolic machinery in brain cortical cells adapts to ensure the maintenance of the energy supply necessary for cell functioning. It is noteworthy that we did not notice any significant alteration in the activation status of AMPK (AMP-activated protein kinase), the cellular energy sensor that acts to restore and/or maintain the energy balance within the cell, as well as the ratio of AMP/ATP (Supplementary Data).

### 2.5. Brain Cortical Mitochondria Biogenesis Is Altered by Long-Term Hyperglycemia and Recurrent Hypoglycemia

**Figure 4:**
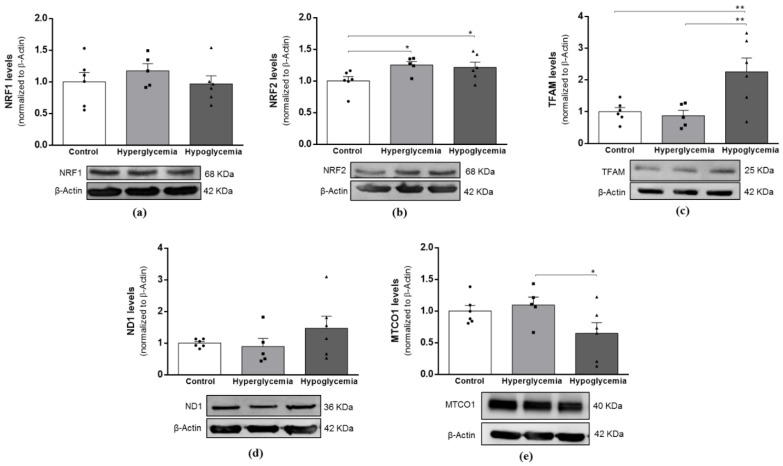
Effects of long-term hyperglycemia and insulin-induced recurrent hypoglycemia on mitochondrial biogenesis and protein levels of mitochondrial genes. Western blot analyses of (**a**) NRF1, (**b**) NRF2, (**c**) TFAM, (**d**) ND1 and (**e**) MTCO1 protein levels. Bars represent the mean ± SEM of the quantification of brain cortical samples from 5-6 rats under each condition after normalization with β-Actin levels with respect to control group. Representative immunoblots for each protein and the respective β-Actin results are shown below the graphs (for uncropped images of the Western blots, please see Supplementary Data). ** *p* < 0.01 and * *p* < 0.05 were considered statistically significant differences.

Extensive evidence suggests a role for mitochondrial dysfunction in the pathophysiology of diabetes-related glycemic variations, hyperglycemia, hypoglycemia and glucose-fluctuations. To substantiate our study, Western blot analysis was used to assess the protein content of core markers of mitochondrial biogenesis, as well as of important mitochondria DNA (mtDNA)-encoded subunits of respiratory chain complexes, the NADH dehydrogenase subunit 1 (ND1) and the mitochondrial-encoded cytochrome c oxidase 1 (MTCO1), which are subunits of respiratory complexes I and IV, respectively. As shown in Figure 4, both metabolic insults significantly increased the protein levels of NRF2 (Figure 4b), which is a well-known transcription factor involved in coordinating antioxidant response to oxidative stress. Furthermore, the exposure to recurrent hypoglycemic episodes evoked a significant increase in TFAM protein levels (Figure 4c) and a decrease in the MTCO1 protein content (Figure 4e), implying an adaptation of brain cortical mitochondria to dysglycemic insults.

### 2.6. Mitochondrial Fission–Fusion Protein Content Is Differently Impacted under Long-Term Hyperglycemia and Recurrent Hypoglycemia

**Figure 5:**
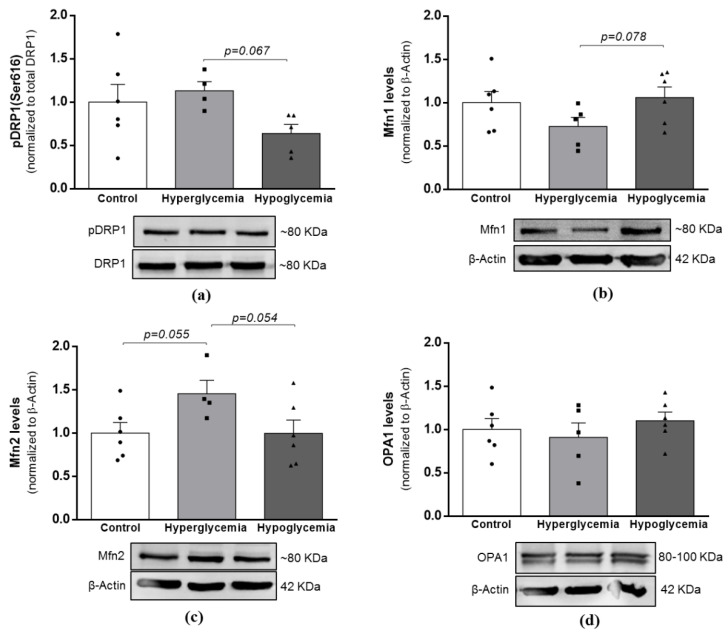
Effects of long-term hyperglycemia and insulin-induced recurrent hypoglycemia on mitochondria dynamics. Western blot analyses of (**a**) pDRP1(Ser616)/DRP1, (**b**) Mfn1, (**c**) Mfn2 and (**d**) OPA1 protein levels. Bars represent the mean ± SEM of the quantification of brain cortical samples from 4-6 rats under each condition after normalization with β-actin levels with respect to control group. Representative immunoblots for each protein and their respective β-Actin results are shown below the graphs (for uncropped images of the Western blots, please see Supplementary Data). (p)DRP1—(phosphorylated) dynamin-related protein 1; Mfn—Mitofusin; OPA1—Optic atrophy 1.

Through the action of specialized proteins, optic atrophy 1 (Opa1) and mitofusin (Mfn) 1 and 2 for fusion and dynamin-related protein 1 (DRP1) for fission, mitochondria can be found either in an interconnected mitochondrial network or in a small, rounded shape, which increases the mitochondrial number or allows the segregation of damaged mitochondria for degradation via mitophagy [22]. Here, the densitometric analysis of blots revealed a trended increase in the protein levels of Mfn2 in the hyperglycemic group (Figure 5c), whereas recurrent hypoglycemia tended to decrease the activation of mitochondrial fission protein pDRP1 when compared to the hyperglycemic animals (Figure 5a). Even though the differences did not reach statistical significance, these observations suggest that brain cortical mitochondria adapt their dynamic processes and change their morphology depending on the metabolic insult.

### 2.7. Recurrent Hypoglycemia Blunts Insulin Signaling-Mediated Pathways Leading to the Activation of GSK3β

**Figure 6:**
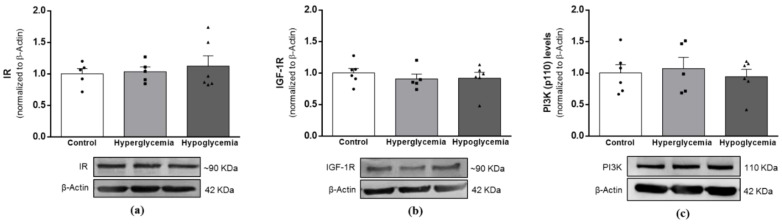
Effects of long-term hyperglycemia and insulin-induced recurrent hypoglycemia on the first steps of insulin signaling. Western blot analyses of (**a**) IRβ, (**b**) IGF1Rβ and (**c**) P13K (p110α) protein levels after normalization with β-actin. Bars in graphs represent the mean ± SEM of the quantification of brain cortical samples from 5–6 rats under each condition. Representative immunoblots for β-Actin are shown as the loading control (for uncropped images of the Western blots, please see Supplementary Data). IR—Insulin receptor; IGF-1R—Insulin-like growth factor 1 receptor; PI3K (P110)—catalytic subunit of phosphoinositide 3 kinase.

**Figure 7:**
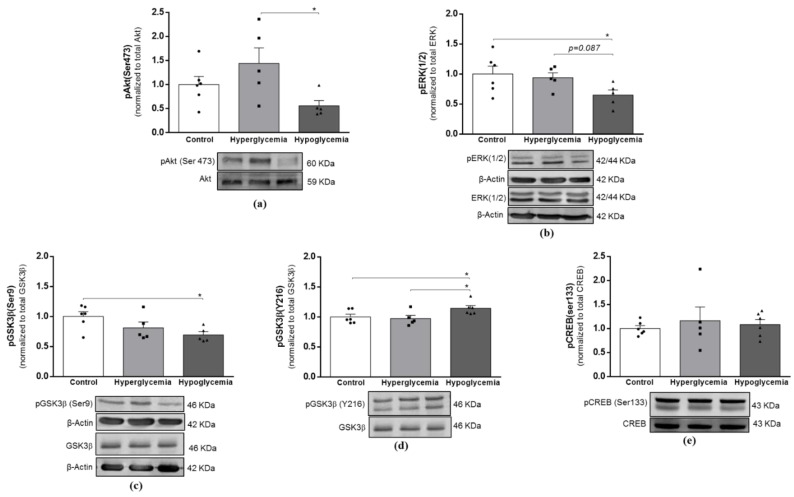
Effects of long-term hyperglycemia and insulin-induced recurrent hypoglycemia on insulin-mediated downstream signaling pathways. Western blot analyses of the ratio of (**a**) pAkt/total Akt, (**b**) pERK/total ERK, (**c**) pGSK3β(Ser9)/total GSK3β, (**d**) pGSK3β(Y216)/total GSK3β and (**e**) pCREB(Ser133)/total CREB. Graphs show mean ± SEM of the quantification of brain cortical samples from 5-6 rats under each condition normalized to control animals. Representative immunoblots are shown below the graphs (for uncropped images of the Western blots, please see Supplementary Data). * *p* < 0.05 was considered as a statistically significant difference. (p)ERK—(phosphorylated) extracellular signal-regulated kinase; (p) GSK3β—(phosphorylated) glycogen synthase kinase 3β; (p) CREB—(phosphorylated) cAMP-response element binding protein.

Insulin/IGF-1 signaling is believed to be intricately linked to mitochondrial function to empower functional brain signaling and metabolism, with a key role in neuronal survival and memory [23]. To further ascertain more about long-term hyperglycemia and recurrent hypoglycemia-induced changes in insulin-linked signaling activities in brain cortical tissue, we decided to evaluate the protein levels of insulin (IR) and IGF-1 receptor (IGF-1R); the catalytic subunit of PI3K; and the P13K (p110) protein (Figure 6), and the activation status of its canonical downstream mediators, the PI3K/Akt and mitogen-activated protein kinase (MAPK/ERK1/2) pathways (Figure 7). Our results indicate that similarly to the GLUT4 content (Figure 1c), the beginning of the insulin cascade remained unaffected, with no alterations detected in the protein content of IR (Figure 6a), IGF-1R (Figure 6b) or P13K (p110) (Figure 6c). On the other hand, both downstream signaling pathways were found to be compromised in the recurrent hypoglycemic group. As demonstrated, a lower ratio of pAkt/Akt (Figure 7a) and pERK/ERK (Figure 7b) was found in brain cortical tissue from hypoglycemic animals when compared to the hyperglycemic group.

Among other functions, Akt kinase activation is known to be directly involved in the phosphorylation of glycogen synthase kinase 3β (GSK3β) at Ser9, inhibiting its activity [24]. Accordingly, we found that the brain cortical tissue from hypoglycemic animals has decreased levels of pGSK3β (Ser9) (Figure 7c), as well as a significant increase in the pGSK3β (Y216)/GSK3β ratio (Figure 7d), which represents the active form of the enzyme. Of note, total GSK3β levels were not found to be significantly altered (Supplementary Data). In the brain, GSK3β regulates many aspects of cellular structure, function and survival, and its activity has been associated with the compromise of functional outcomes in different models of disease, including diabetes [24,25,26]. Herein, despite the activation of GSK3β, we did not detect any alterations in the activation status of pCREB (Figure 6e), a transcription factor widely implicated in neuronal survival and memory formation [27] and a downstream target of GSK3β. These data suggest that despite the increased activation of GSK3β, brain cortical synaptic function is not compromised under the current protocol of diabetes and recurrent hypoglycemia.

## 3. Discussion

Our findings demonstrate that the brain, namely, the cortex, responds to insulin-induced recurrent hypoglycemia in the context of long-term hyperglycemia by evoking several adaptive metabolic responses. In particular, we observed an elevation in the activity of key enzymes involved in the metabolism of glucose, even in the absence of changes in glucose and monocarboxylic transporters protein expression. This suggests an attempt to preserve glucose flux during periods of hypoglycemia, and may indicate the existence of hypoglycemia unawareness in which glucose continues to be provided to the brain even in the face of systemic hypoglycemia. It was also observed that both metabolic insults promoted distinct alterations in insulin downstream canonical pathways and mitochondrial phenotypes, which eventually represent an attempt to sustain brain cell integrity and function. These results are comparable with, and extend, previous studies reporting that metabolic alterations are one of the key mechanisms mediating hypoglycemia-related brain effects [19].

Under frequent alterations in systemic glucose levels, it is predicted that the glucose availability to the brain is also impacted [28]. In T1D subjects, intensive insulin therapy continues to be the gold standard to attain blood glucose level normalization. However, the benefits of such therapy are largely downsized by the frequent occurrence of bouts of hypoglycemia, a situation that on average occurs more than twice a day in T1D individuals [29], contributing to blunt the brain’s capacity to detect hypoglycemia and activate counter-regulatory mechanisms to correct the reduction in blood glucose levels. From a mechanistic point of view, earlier studies suggest that alterations in brain glucose uptake/transport and metabolism [30,31] may be key contributors to this hypoglycemia unawareness. During different insults of hypoglycemia, both increases [32,33,34] and a lack of alterations [20] in insulin-independent glucose transporter abundance (GLUT1 and GLUT3) have been described. It was also reported that even though the plasma concentration of insulin in diabetic patients may increase due to exogenous insulin bouts or insufficient insulin clearance, such rise does not account for differences in the brain glucose concentration [35]. Additionally, patients with T1D with sufficient exposure to recurrent hypoglycemia to create impaired hypoglycemia unawareness did not show alterations in the hippocampal glucose metabolism [36]. Hyperglycemia seems to have divergent effects in diabetic human subjects and animal models, presenting lower or unchanged brain glucose levels compared to control subjects [37,38], a condition plausibly attributed to a reduction in intracellular glucose transport [39,40]. Similarly, in this study, we did not detect any significant alterations in ether insulin-unresponsive or -responsive glucose transporters in brain cortical lysates from recurrent hypoglycemic animals, but a significant decrease in GLUT3 content was found in hyperglycemic rats. These observations support the notion that brain glucose transport uptake is directly modulated in response to plasma glucose, and that different types of metabolic insults likely have divergent effects on the brain glucose transport machinery [37]. However, we must emphasize that we did not assess the activity and/or the location of GLUTs and/or MCT2, and functional studies are required to clarify this issue.

The major pathways of the brain glucose metabolism comprise glycolysis and Krebs cycle, which together are responsible for generating the majority of the ATP required to support brain functions (for review, see ref. [41]). Our study shows that from the initial steps of glucose metabolism to the Krebs cycle, recurrent hypoglycemia induces an up-regulated and continued metabolism of glucose in brain cortical samples. This adaptation may be likely to preserve glucose flux during recurrent hypoglycemia, but at the same time, can interfere with mechanisms of hypoglycemia detection and counter-regulation [42]. Regarding hyperglycemia, the available data are scarce, and some suggest that hyperglycemia induces a compensatory metabolic shift via mitochondrial complex II [43]. It was observed that the rate of G6P synthesis was higher in the diabetic group than in the control animals [43]. Aconitase and α-KGDH activities are sensitive to the presence of reactive oxygen species (ROS); however, whilst aconitase becomes dysfunctional, α-KGDH continues to be functional, as long as the NADH supply is maintained [44]. In close agreement with, alongside a decreased aconitase activity [13], our mitochondrial preparations from hyperglycemic animals also presented a trended increment in α-KGDH activity. We can speculate that the TCA cycle in hyperglycemic animals is truncated at the aconitase steps, but there is an attempt to maintain the functionality of this pathway from α-KGDH onwards, as we did not notice alterations in SDH activity. In agreement with [45], a similar situation may occur in hypoglycemic rats, in which aconitase activity is also decreased [13], but the activities of PDH, α-KGDH and SDH are increased.

In parallel with the catabolic pathways, it has been demonstrated that glucose in the neurons can be redirected into the pentose phosphate pathway (PPP) to maintain the neuronal antioxidant status at the expense of glucose utilization for energy production [46]. Localized in the cytosol, G6PD is the first rate-limiting enzyme of the PPP, in which G6P is oxidized to produce the reduced form of nicotinamide adenine dinucleotide phosphate (NADPH), which is then required for the glutathione system and other ROS scavengers to preserve cellular redox homeostasis [47]. Previous studies report an increase in the activity of brain G6PD in response to increased pro-oxidant activity [48,49]. A decrease in the levels of glycolysis and PPP substrates during hypoglycemic coma was previously reported [50]. Herein, G6PD activity decreased significantly in response to both metabolic insults. These changes could correlate with the previously assessed brain oxidative status [13], characterized by a decrease in the GSH/GSSG ratio, increased glutathione reductase activity, and unchanged glutathione peroxidase activity and H_2_O_2_ levels. It is noteworthy that we also found a significant increment in the protein content of NRF2 in both metabolic conditions. Compelling data indicate that NRF2 is a transcription factor involved in regulating cellular antioxidant defenses, as well as coordinating cytoprotective responses to oxidative stress [51]. In fact, some studies report the potential of targeting the Nrf2-dependent defense mechanism when designing therapeutic strategies to prevent both hyperglycemia- and hypoglycemia-mediated effects [52]. We can, therefore, infer that, under our experimental conditions, glucose/G6P is not directed to PPP; instead, it is mainly channeled to support energy production.

The inherent need of the brain for a high energy supply dictates its dependence on functional mitochondria but also renders it sensitive to changes in these organelles. Mitochondrial homeostasis greatly depends on the interplay between mitochondrial biogenesis and fission/fusion cycles. Alterations in these processes have been frequently proposed to be responsible for mitochondrial dysfunction in neurodegenerative disorders and diabetes [15,53]. A previous study from our laboratory showed that mitochondrial biogenesis, through increased levels of NRF2, TFAM and mtDNA copy number, occurs in the brain cortex of T1D animals [12], probably representing a compensation mechanism to prevent alterations in mitochondrial function and the activation of apoptosis. Recurrent hypoglycemia under diabetic conditions caused morphological alterations of the hippocampal mitochondria, mainly due to an increased mitochondrial fission, which underlined synapse injury and cognitive deficits [15]. Other studies showed that both low- [54] and high-glucose exposure induces the loss of the mitochondrial network via increased Drp1 activation and consequent mitochondrial fragmentation in endothelial cells [55]. In contrast, we showed a trended decreased activation of Drp1 and increased levels of TFAM in recurrent hypoglycemic animals, whilst in diabetic animals, the levels of the fusion protein Mfn2 presented a trended increase, and Mfn1 showed a trended decrease. In sum, these findings, coupled with the abovementioned metabolic alterations, may indicate the existence of a coordinated and reciprocal relationship between the functional metabolism and mitochondria phenotype under different glycemic insults. Thus, the increased mitochondrial biogenesis and decreased fission in response to hypoglycemia possibly point to an attempt to allow cells to face alterations in energy supply, thereby upholding brain cell energy requirements and viability. It is noteworthy that more experiments, such as microscopic analysis, would be helpful to fully elucidate such assumption.

Insulin/IGF-1 and downstream pathways in the brain, besides contributing to glucose metabolism regulation through the up-regulation of GLUT4, are strongly involved in neuronal survival, synaptic plasticity and function. In contrast, their deregulation has been associated with diverse neurologic and neurodegenerative disorders, including Alzheimer’s disease (AD) [56,57,58]. According to some studies, PI3K/Akt/GSK3β represent a link between T1D and the risk of developing neurodegenerative disorders such as AD [26,59]. It was previously shown that brain tissue from STZ-treated rodents presents reduced insulin-signaling pathway activity and increased GSK3β activity, concomitant with impaired cognitive behavior and the appearance of AD hallmarks [26]. Moreover, T1D was shown to exacerbate synaptic damage, learning deficits and AD hallmarks in transgenic mouse models of AD [60,61]. However, others report a lack of changes in the initial and downstream stages of insulin signaling in STZ-treated rodents [12,62]. In this study, hyperglycemia did not affect any of the evaluated steps of insulin cascade. In contrast, recurrent hypoglycemic episodes significantly reduced Akt activation and phosphorylated ERK, promoting the activation of GSK3β in brain cortical tissue. Accordingly, a previous in vitro study showed that glucose deficiency-mediated effects are associated with ERK inhibition [63] and the decreased activation of Akt [64], concomitantly with the increased activation of GSK3β. However, and unlike other studies reporting a negative effect of hypo- and hyperglycemia on learning and memory processes [65], we did not detect any alteration in the CREB activation status, suggesting the preservation of brain cortex-dependent functions. In support of this, previous studies from our laboratory also showed that hypo- and hyperglycemia do not affect the levels of the synaptic marker synaptophysin in the brain cortex [12,13].

It must be considered that the variability in the degree and duration of hyperglycemia, as well as of the type of hypoglycemic insult, may account for the discrepancy among different studies. Additionally, the background of hypoglycemia, i.e., whether it occurs in a diabetic or control context, seems to be an important confounder variable when interpreting different data [37]. Noteworthy, an important limitation of our experimental setting is the fact that it did not allow us to distinguish the effects occurring in the different cellular populations, namely, neurons and astrocytes. It was previously shown that astrocytes have the ability to supply neurons with other energy substrates to sustain neuronal metabolism during hypoglycemia [20]. In fact, even though we did not detect alterations in MCT2 protein levels and did not measure the levels of alternative substrates, the possible contribution of alternative sources of energy to maintaining the brain metabolism and energetics under hypoglycemic conditions cannot be ignored. Further investigation is necessary to elucidate this matter.

In summary, in the present study, we uncovered some of the mechanisms by which the brain responds to different glycemic situations, providing experimental evidence that insulin-induced recurrent hypoglycemia has the capacity to evoke adaptive responses in the brain cortex. Understanding the relationship among the metabolism, mitochondria homeostasis and signaling pathways is essential to decipher the response of brain cells and anticipate the functional outcomes induced by different metabolic insults.

## 4. Materials and Methods

### 4.1. Chemicals and Reagents

Streptozotocin (STZ) was obtained from Sigma (Portugal). Insulin (Humulin NPH) was obtained from Eli Lilly and Company (USA). The primary antibodies used for Western blotting were obtained from the following sources: Actin (1:5000, Sigma (A5441)); Akt (1:1000, BD Biosciences (610861)); pAkt (Ser473) (1:1000, Cell Signaling (4051)); AMPKα (1:1000, Cell Signaling (2532)); pAMPKα (Thr172) (1:1000, Cell Signaling (2535)); CREB (1:750, Cell Signaling (9197)); pCREB(Ser133) (1:1000, Millipore (06-519)); DRP1 (1:1000, BD Biosciences (611113)); pDRP1(Ser616) (1:1000, Cell Signaling (3455)); MAPK (ERK1/2) (1:1000, Cell Signaling (9102)); pMAPK (ERK1/2) (Thr202/Tyr204) (1:1000, Cell Signaling (4377)); pGSK3β (Ser9) (1:1000, Cell Signaling (9336)); pGSK3β (Y216) (1:1000; Santa Cruz Biotechnology (sc-135653)); GSK3β (1:1000, Santa Cruz Biotechnology (sc-81462)); GLUT1 (1:1000, Millipore (CBL242)); GLUT3 (1:1000, Abcam (ab41525)); IGF-1R β(H60) (1:1000, Santa Cruz Biotechnology (sc-9038)); IR β(4B8) (1:1000, Cell Signaling (3025)); ND1 (C-18) (1:1000, Santa Cruz Biotechnology (sc-20493)); NRF1 (1:1000, Santa Cruz Biotechnology (sc-33771)); NRF2 (1:1000, Abcam (ab31163)); TFAM (1:750, Santa Cruz Biotechnology (sc-23588)); MCT2 (1:1000, Santa Cruz Biotechnology (sc-14926)); Mfn1 (1:1000, Santa Cruz Biotechnology (sc-50330)); Mfn2 (1:1000, Santa Cruz Biotechnology (sc-100560)); MTCO1 (1:1000, Abcam (ab14705)); OPA1 (1:1000, BD Biosciences (612607)); PI3K (p110-alpha-C73F8) (1:1000, Cell Signaling (4249S)); PSD95 (1:1000, Cell Signaling (D27E11)); SDHA (1:1000, Cell Signaling (5839)); pTau (Ser 396) (1:1000, Santa Cruz Biotechnology (sc-101815)); Tau (1:750, Cell Signaling (4019). All other chemicals were of the highest grade commercially available purity, and all aqueous solutions were prepared in ultrapure (type I) water.

### 4.2. Treatment of Animals

Eighteen male Wistar rats (2 months old) from Charles River were housed at the Center for Neuroscience and Cell Biology (CNC) Animal House Facility (license n° 520.000.000.2006, from the Portuguese Animal Welfare authorities) and maintained under controlled light (12 h day/night cycle), temperature (22–24 °C) and humidity (50–60%) conditions with free access to water and powdered rodent chow (except in the fasting period). The day before injections, rats were randomly divided in two groups and were deprived of food overnight. The next day, one group received an intraperitoneal (i.p.) injection of STZ (50 mg/kg body weight) freshly dissolved in citrate 100 mM, pH 4.5. The volume administered was always 0.5 mL/200 g body weight. The control group received an i.p. injection with an equal volume of citrate (vehicle). In the following 24 h, animals were given free access to glycosylated water in order to avoid hypoglycemia resulting from the massive destruction of β-cells and the release of intracellular insulin associated with STZ treatment [13]. Three days after STZ administration, the tail vein blood glucose levels were measured in all animals, and those presenting levels above 250 mg/dL were considered diabetic. Control and chemically induced diabetic rats were maintained for 3 months with the regular monitoring of the animals’ welfare, blood glucose and body weight; then, a cohort of diabetic rats were subjected to recurrent hypoglycemia induced by twice-daily subcutaneous injections of intermediate-acting insulin with dosage adjustment according to the blood glucose levels over two weeks [13]. All procedures were performed to minimize exposure to stress and suffering, in accordance with the animal welfare guidelines of the local Animal Welfare and Ethics Body (Project ORBEA_61_2013/24072013), the Federation of European Laboratory Animal Science Associations (FELASA) and Portuguese legislation (Directive 2010/63/ EU; DL113/2013, 7 August).

### 4.3. Determination of Blood Glucose and Glycated Hemoglobin Levels

The blood glucose concentration was determined from the tail vein using a commercial glucometer (Glucometer-Elite, Bayer, Portugal). Hemoglobin A1C (HbA1c) levels were determined using Systems SYNCHRON CX 4 (Beckman). This system utilizes two cartridges, Hb and A1c, to determine A1c concentration as a percentage of total Hb. The hemoglobin was measured using a colorimetric method and the A1c concentration by a turbidimetric immunoinhibition method. Both parameters were measured under non-fasting conditions.

### 4.4. Preparation of Mitochondrial Fraction

Brain cortical mitochondria were isolated from rats as previously described [13,14]. In brief, after animals were sacrificed, the brains were rapidly removed, and the cerebral cortexes were dissected on ice and homogenized at 4 °C in 10 mL of isolation medium (225 mM mannitol, 75 mM sucrose, 5 mM HEPES, 1 mM EGTA, 1 mg/mL BSA, pH 7.4) containing 5 mg of bacterial protease type VIII (Subtilisin). Single brain homogenates were brought to 20 mL and then centrifuged at 2500 rpm (Sorvall RC-5B Refrigerated Superspeed Centrifuge) for 5 min. The pellet, including the fluffy synaptosomal layer, was resuspended in 10 mL of isolation medium containing 0.02% digitonin and centrifuged at 10,000 rpm for 10 min. The brown mitochondrial pellet without the synaptosomal layer was then resuspended again in 10 mL of medium and centrifuged at 10,000 rpm for 5 min. The pellet was resuspended in 10 mL of washing medium (225 mM mannitol, 75 mM sucrose, 5 mM HEPES, pH 7.4) and centrifuged at 10,000 rpm for 5 min. The final mitochondrial pellet was resuspended in the washing medium, and the mitochondrial protein was determined by the biuret method calibrated with BSA (13).

### 4.5. Brain Tissue Processing for Enzymatic Activity Determination

After the sacrifice of the animals, the brains were quickly removed, and the cerebral cortexes were dissected on ice, flash-frozen using liquid nitrogen and stored at −80 ° C. At the time of the experiments, a small part of the cortical tissue was homogenized in phosphate buffer (pH 7.4), and protein determination was performed by the BCA protein assay using the BCA kit (Pierce Thermo Fisher Scientific, Rockford, IL, USA).

### 4.6. Measurement of Hexokinase Activity

Hexokinase activity was determined in brain cortical homogenates or in isolated mitochondria to separately determine the activity of hexokinase I localized in mitochondria and of hexokinase II, which is localized in the cytoplasm [19]. Briefly, 300 µg of cortical extracts or 100 µg of isolated mitochondria was added to the reaction medium (10 mM MgCl2, 216 mM glucose, 1.2 mM nicotinamide adenine dinucleotide phosphate (NADP^+^), supplemented with 1.1 mM ATP, and 2 U glucose-6-phosphate dehydrogenase in 50 mM Tris–HCl (pH 8)). The reaction was initiated with the addition of 216 mM glucose and was continuously measured for 3 min at 340 nm, 25 °C (ε = 6.22 mM^−1^cm^−1^) [66].

### 4.7. Measurement of Glucose-6-Phosphate Dehydrogenase Activity

Glucose-6-phosphate dehydrogenase activity was determined by measuring the rate of NADPH production according to Lohr and Waller [67]. Briefly, brain cortical homogenates (25 μg) were added to the reaction medium (5 mM MgCl_2_, 0.3 mM NADP, and 10 mM glucose-6-phosphate in 50 mM Tris-HCl (pH 7.5)). NADPH production was continuously monitored at 340 nm, 25 °C (ε = 6.22 mM^−1^cm^−1^).

### 4.8. Measurement of Pyruvate Dehydrogenase (PDH) Activity

PDH activity was measured spectrophotometrically, as described previously [68]. In brief, brain cortical mitochondria (100 μg) were added to the reaction medium (35 mM KH_2_PO_4_, 5 mM MgCl_2_, 2 mM KCN, 0.5 mM EDTA, 0.25% X-Triton, pH 7.2) supplemented with 2.5 μM rotenone, 0.5 mM of NAD^+^, 0.2 mM thiamine pyrophosphate (TPP), 0.13 mM coenzyme A (CoA), and 1 mM cysteine. The reaction was started by the addition of 4 mM sodium pyruvate followed by 340 nm at 30 °C (ε = 6.22 mM^−1^cm^−1^).

### 4.9. Measurement of Alpha-Ketoglutarate Dehydrogenase (A-KGDH) Activity

For α-KGDH activity, brain cortical mitochondria (100 μg) were added to the reaction medium (25 mM KH_2_PO_4_, 5 mM MgCl_2_, 2 mM KCN, 0.5 mM EDTA, 0.25% X-Triton, pH 7.25) supplemented with 10 mM CaCl_2_, 2.5 μM rotenone, 0.2 mM of NAD^+^, 0.3 mM TPP, 0.13 mM CoA and 1 mM cysteine [69]. The reaction was started by adding 5 mM of α-ketoglutarate, followed by the reduction of NAD+ at 30 °C (ε = 6.22 mM^−1^cm^−1^).

### 4.10. Measurement of Succinate Dehydrogenase Activity

Succinate dehydrogenase activity was assayed by adding brain cortical mitochondria (100 μg) to the reaction medium (100 mM Tris-HCl pH 7.5) supplemented with 0.5 mM EDTA, 2 μM antimycin A, 2 μM rotenone, 10 mM sodium azide and 0.25 mM nitroblue tetrazolium (NBT). The reaction was started with 20 mM succinate, and the absorbance was monitored at 500 nm at 37 °C for 60 min (ε = 21 mM^−1^cm^−1^) [70].

### 4.11. Measurement of Citrate Synthase Activity

To evaluate mitochondrial citrate synthase activity, 50 µg of brain cortical mitochondria was added to 100 mM Tris-HCl (pH 8) reaction buffer containing 0.1% Triton X-100, 200 µM 5,50-dithiobis-2-nitrobenzoic acid (DTNB) and 200 µM acetyl-CoA, and the baseline was measured at 412 nm for 3 min with 30s intervals, at 30 °C. The reaction was started with 100 µM oxaloacetate and followed spectrophotometrically in a SpectraMax Plus 384 microplate reader for 6 min by measuring the increase in absorbance at 412 nm (=13.6 mM/cm) resulting from the reduction of DTNB. Results were expressed as nmol/min/mg protein.

### 4.12. Determination of Adenine Nucleotide Levels

Adenine nucleotide levels were determined using brain cortical mitochondria after the determination of oxidative phosphorylation parameters [13]. Briefly, at the end of each mitochondrial membrane potential measurement, 250 μL of each sample was promptly centrifuged at 14,000 rpm (Eppendorf centrifuge 5415C) for 2 min with 250 μL of 0.3 M perchloric acid (HClO_4_). The supernatants were neutralized with 10 M KOH in 5 M Tris and again centrifuged at 14,000 rpm for 2 min. The resulting supernatants were assayed for adenine nucleotide by separation using reverse-phase high-performance liquid chromatography (HPLC). The HPLC apparatus was a Beckman-System Gold, consisting of a 126 Binary Pump Model and 166 Variable UV detector controlled by a computer. The detection wavelength was 254 nm, and the column was a Lichrospher 100 RP-18 (5 µm) from Merck. An isocratic elution with 100 mM phosphate buffer (KH_2_PO_4_; pH 6.5) and 1.2% methanol was performed with a flow rate of 1 mL/min. The required time for each analysis was 5 min. Adenine nucleotides were identified by their chromatographic behavior (retention time, absorption spectra and correlation with standards).

### 4.13. Western Blot Analysis

Previously flash-frozen, brain cortical tissue was homogenized in RIPA buffer (150 mM NaCl, 0.1% sodium dodecyl sulfate (SDS), 1% sodium deoxycholate (DOC), 1% nonyl phenoxypolyethoxylethanol (NP-40), 50 mM Tris, pH 7.4) containing 0.1 M PMSF, 0.2 M DTT, and protease and phosphatase inhibitors (Roche Applied Science). The homogenate was incubated on ice for 15 min; frozen and defrozen 3 times to favor disruption; centrifuged at 14,000 rpm (Eppendorf centrifuge 5415C) for 10 min, at 4 °C; and the resulting supernatant was collected and stored at −80 °C. The amount of protein in the samples was analyzed by the BCA protein assay using the BCA kit (Pierce Thermo Fisher Scientific, Rockford, IL, USA). The samples were resolved by electrophoresis in 10–12% SDS–polyacrylamide gels and transferred to polyvinylidene difluoride (PVDF) membranes. After electrophoresis, proteins were transferred to PVDF membranes, and blocked membranes (1 h in 5% BSA and 0.1% Tween in TBS for 1 h at room temperature) were incubated overnight at 4 °C with primary antibodies. The next day, membranes were washed 3 times with 0.1% Tween in TBS and incubated with corresponding secondary antibodies. β-Actin was used as a loading control. The enhanced chemifluorescent (ECF) detection system (GE Healthcare) and Versa Doc or Chemi Doc imaging systems (Bio-Rad, Hercules, CA) were used, and band density was obtained with the Quantity One Software or Image Lab Software, respectively (Bio-Rad).

### 4.14. Statistical Analysis

Results are presented as the mean ± SEM of the indicated number of experiments. After assessing the normality distribution of the groups using GraphPad Prism Software Inc. (La Jolla, CA, USA), data were analyzed using the one-way ANOVA test for multiple comparisons, followed by the post hoc Fisher’s LSD test. A *p* value < 0.05 was considered statistically significant.

## Figures and Tables

**Table 1:** Characterization of the experimental animal models.

Groups	Body Weight (g)	Brain Weight (g)	Glucose (mg/dl)	Hb_A1C_ (%)
Control	442 ± 7.9	1.99 ± 0.03	122.3 ± 2.5	3.6 ± 0.03
Hyperglycemia	280 ± 6.5 ****	1.96 ± 0.05	472.8 ± 28.6 ****	8.5 ± 0.40 ****
Recurrent Hypoglycemia	330 ± 17.7 ****^; ++^	2.10 ± 0.13	44.8 ± 6.8 **^; ++++^	7.1 ± 0.27 ****^; ++^

Data are the means ± SEM of 6 animals under each condition after three months of chemically induced diabetes and two weeks of recurrent hypoglycemia. Glucose and Hb_A1C_ levels were measured under non-fasting conditions. Statistical significance: **** *p* < 0.0001 and ** *p* < 0.01 when compared with control rats; ^++++^ *p* < 0.0001 and ^++^ *p* < 0.01 when compared with STZ-treated rats. Hb_A1C_—glycated hemoglobin.

## Data Availability

The data presented in this study are available in the published article.

## Supplementary Materials

The following are available online at https://www.mdpi.com/article/10.3390/ijms222413470/s1.
