# Supplementary figures and images for: Insulin-Induced Recurrent Hypoglycemia Up-Regulates Glucose Metabolism in the Brain Cortex of Chemically Induced Diabetic Rats
Source: Int J Mol Sci. 2021 Dec 15;22(24):13470. doi: 10.3390/ijms222413470 (PMC8708764; doi:10.3390/ijms222413470)

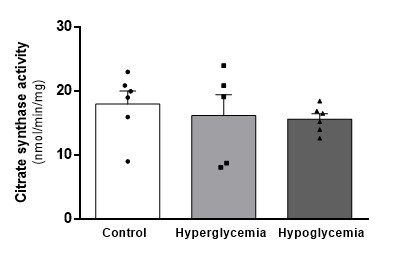

Supplement: Supplementary file 1 [file ijms-22-13470-s001.zip › Figs. Supp/Figure S1.jpg]

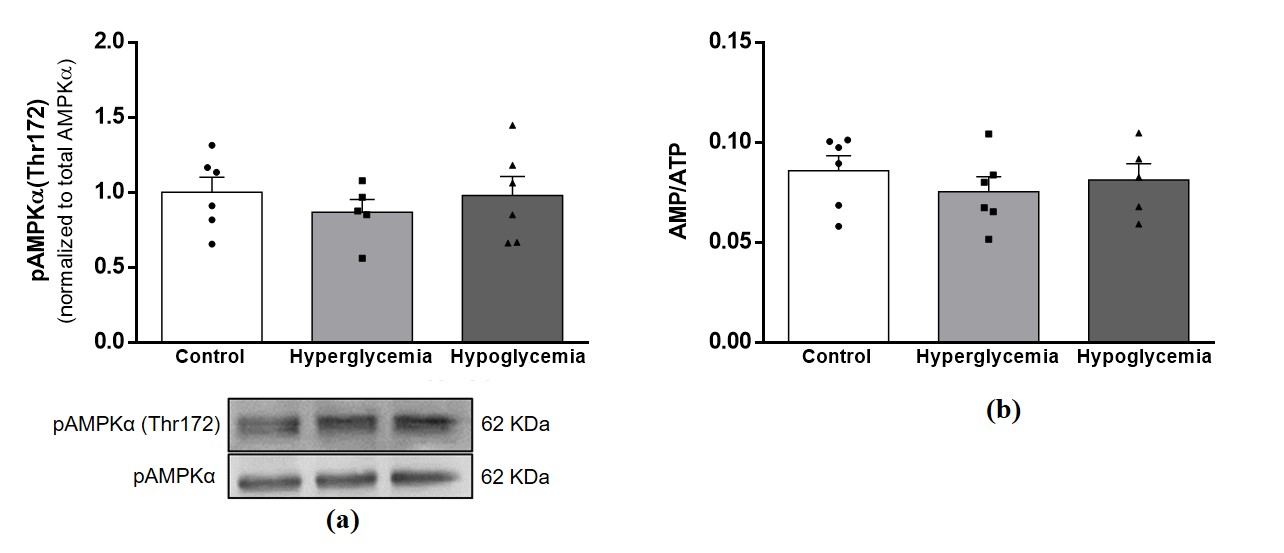

Supplement: Supplementary file 1 [file ijms-22-13470-s001.zip › Figs. Supp/Figure S2.jpg]

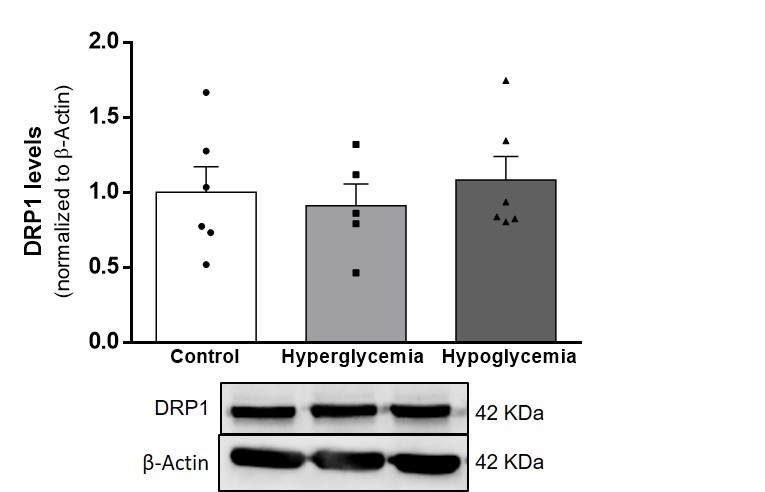

Supplement: Supplementary file 1 [file ijms-22-13470-s001.zip › Figs. Supp/Figure S3.jpg]

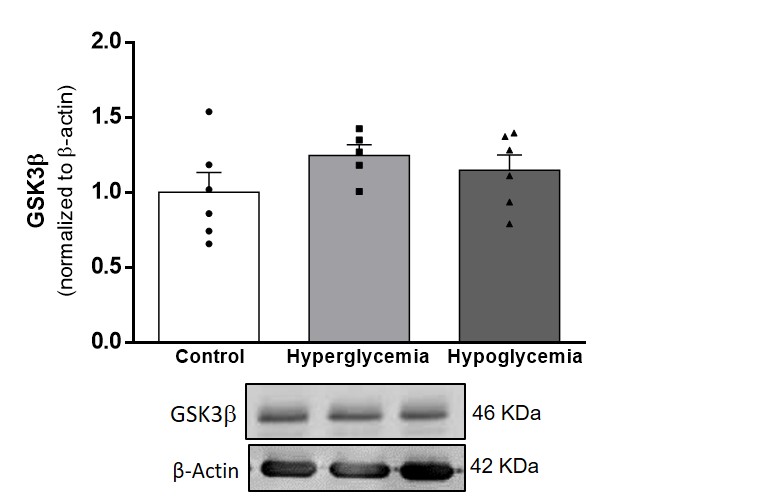

Supplement: Supplementary file 1 [file ijms-22-13470-s001.zip › Figs. Supp/Figure S4.jpg]

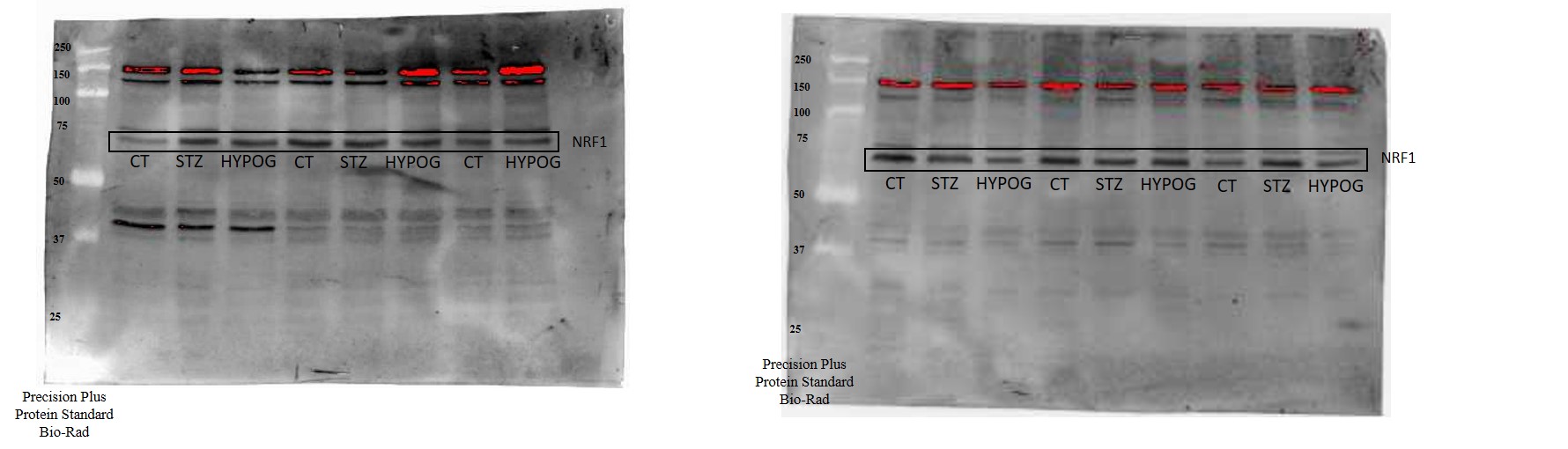

Supplement: Supplementary file 1 [file ijms-22-13470-s001.zip › Figs. Supp/Figure S10.jpg]

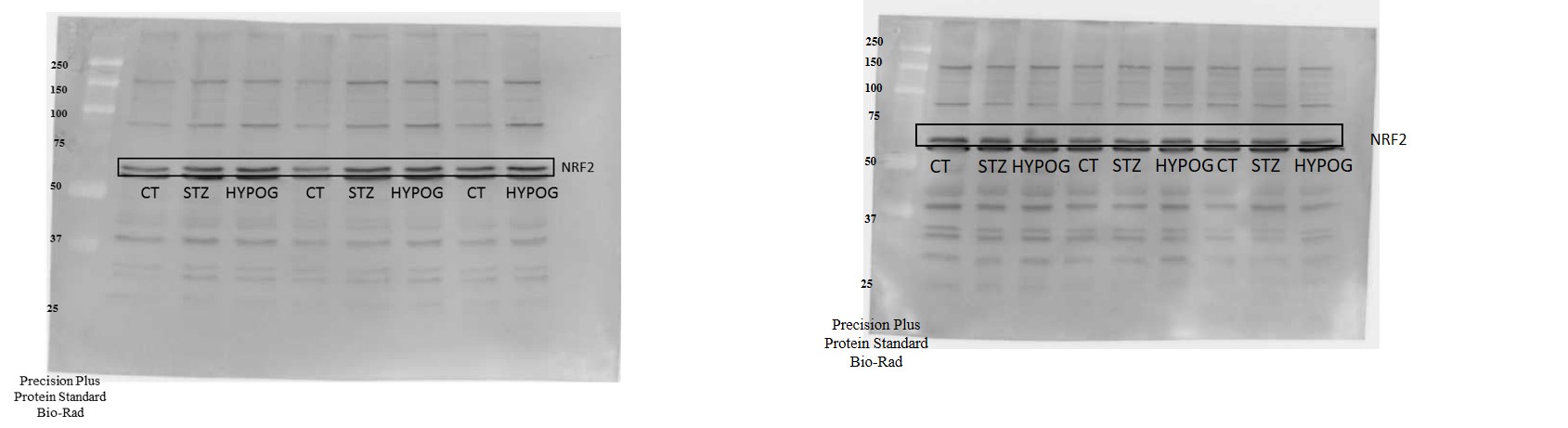

Supplement: Supplementary file 1 [file ijms-22-13470-s001.zip › Figs. Supp/Figure S11.jpg]

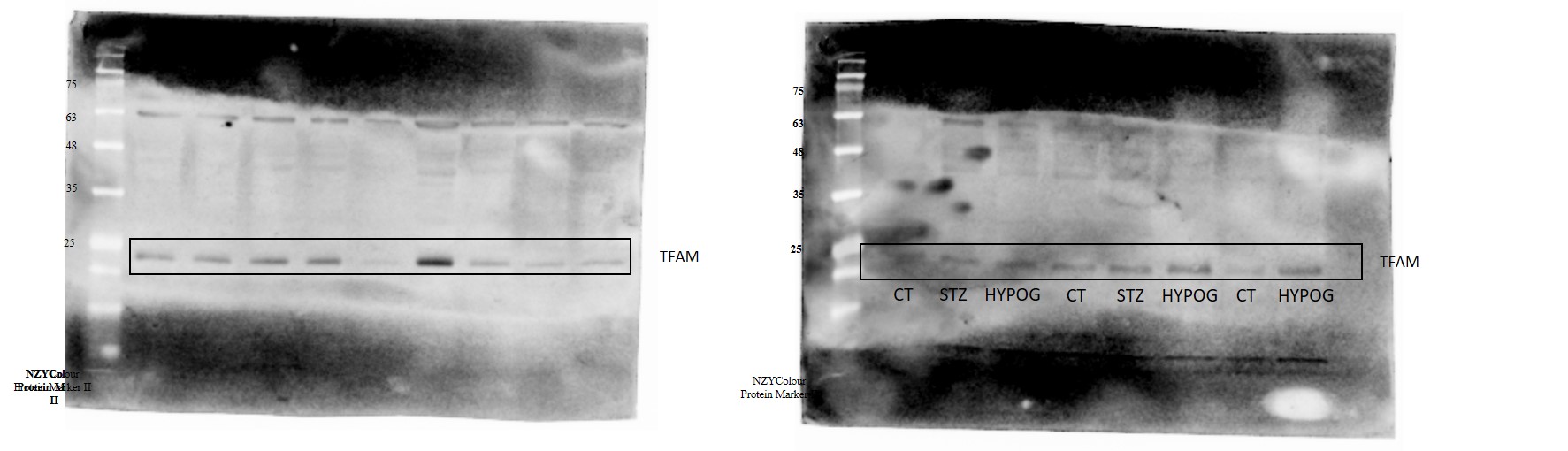

Supplement: Supplementary file 1 [file ijms-22-13470-s001.zip › Figs. Supp/Figure S12.jpg]

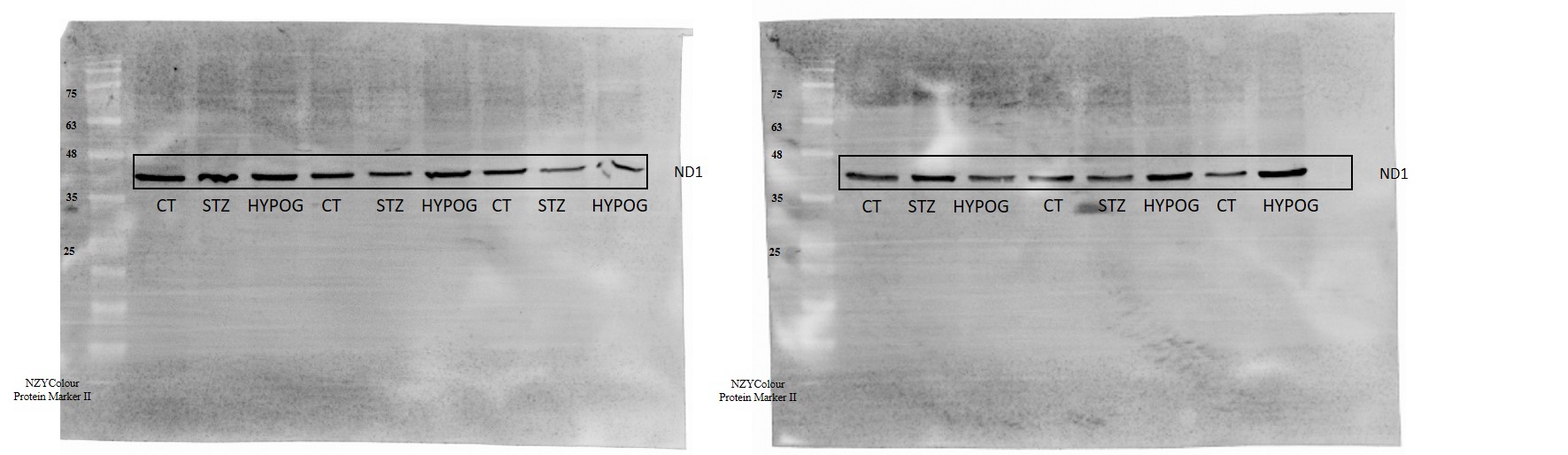

Supplement: Supplementary file 1 [file ijms-22-13470-s001.zip › Figs. Supp/Figure S13.jpg]

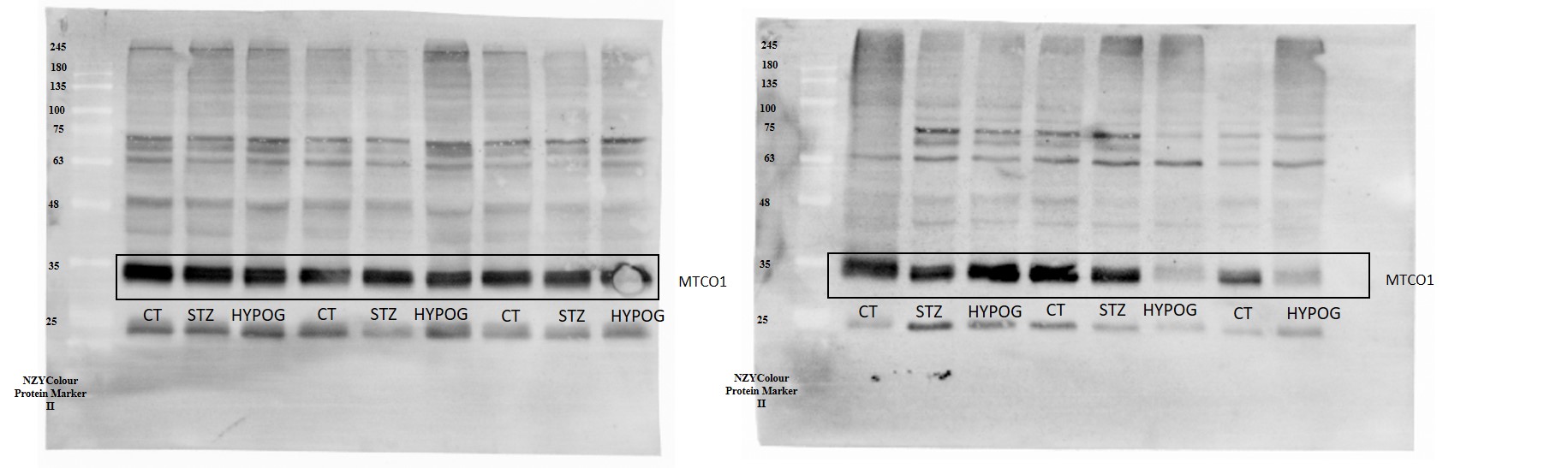

Supplement: Supplementary file 1 [file ijms-22-13470-s001.zip › Figs. Supp/Figure S14.jpg]

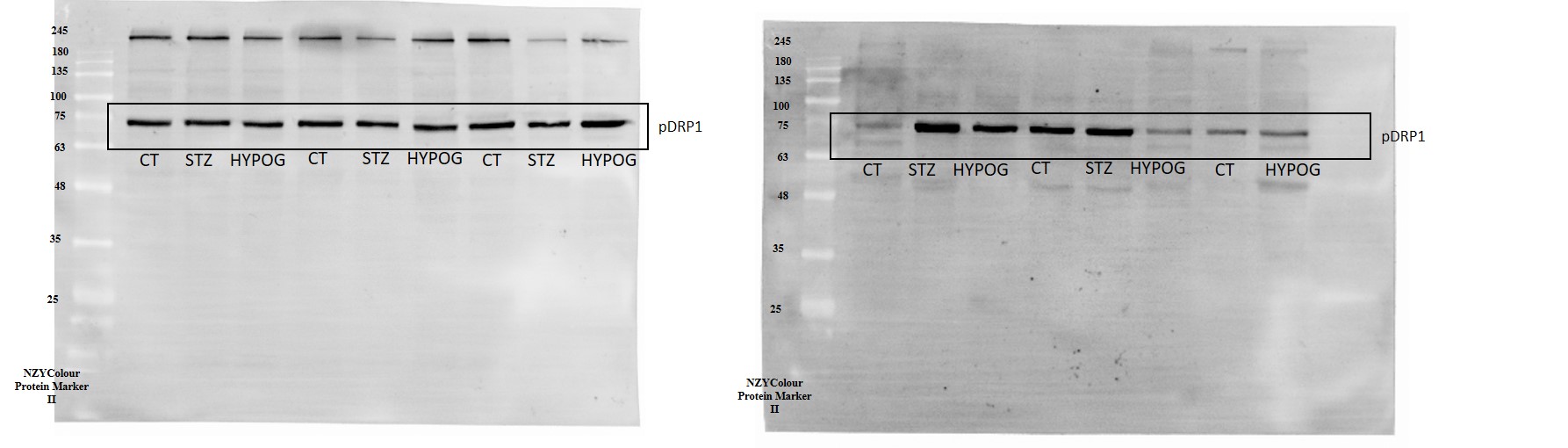

Supplement: Supplementary file 1 [file ijms-22-13470-s001.zip › Figs. Supp/Figure S15.jpg]

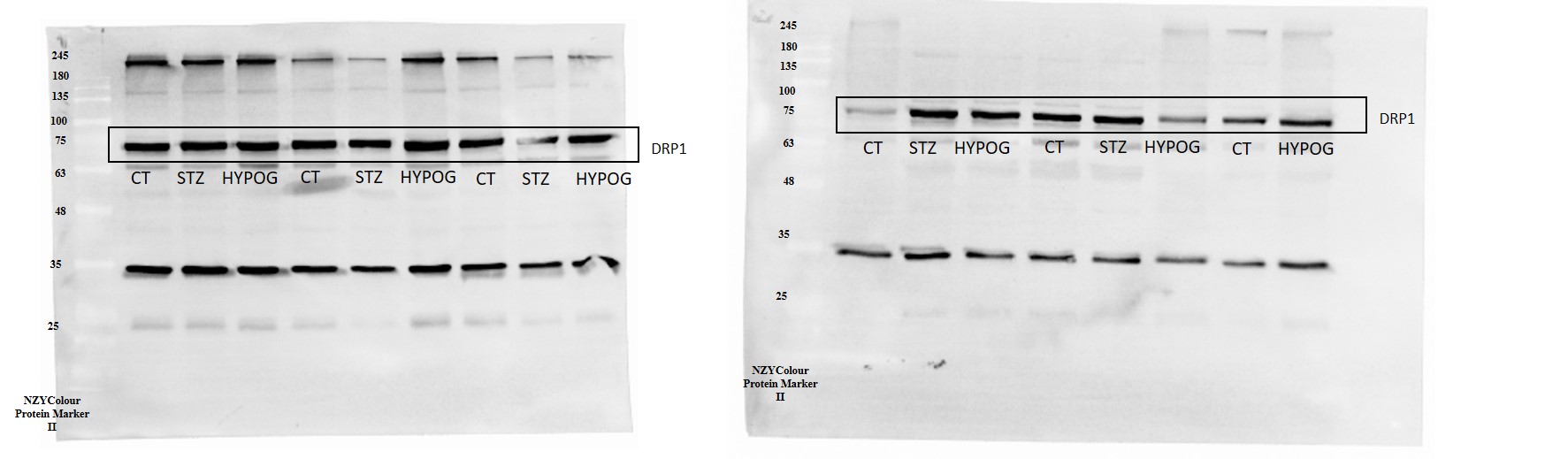

Supplement: Supplementary file 1 [file ijms-22-13470-s001.zip › Figs. Supp/Figure S16.jpg]

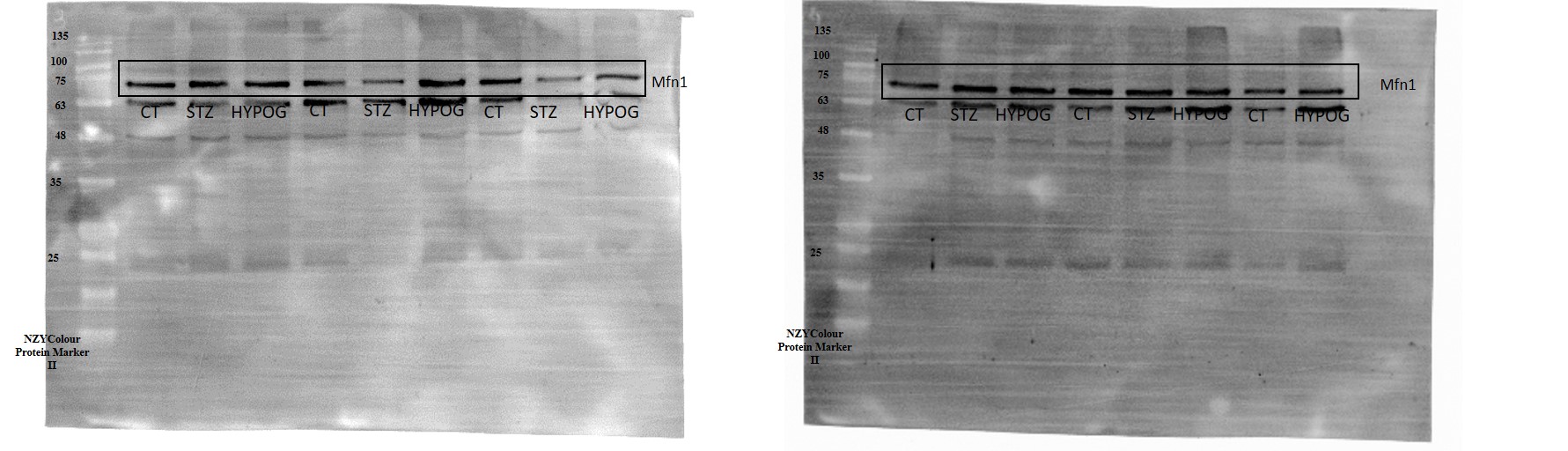

Supplement: Supplementary file 1 [file ijms-22-13470-s001.zip › Figs. Supp/Figure S17.jpg]

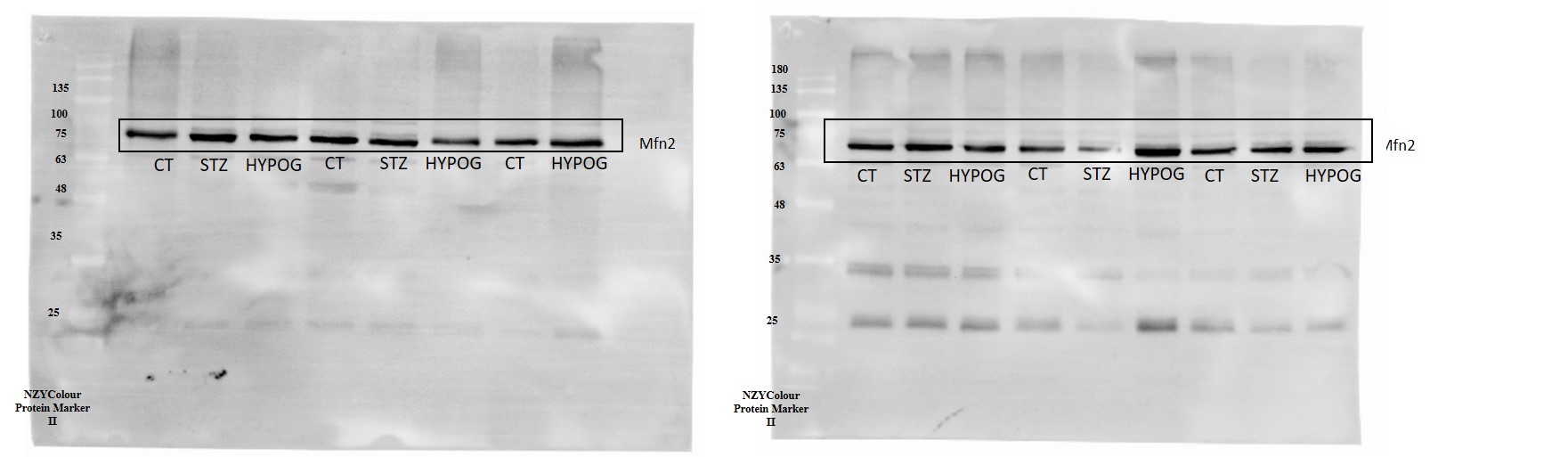

Supplement: Supplementary file 1 [file ijms-22-13470-s001.zip › Figs. Supp/Figure S18.jpg]

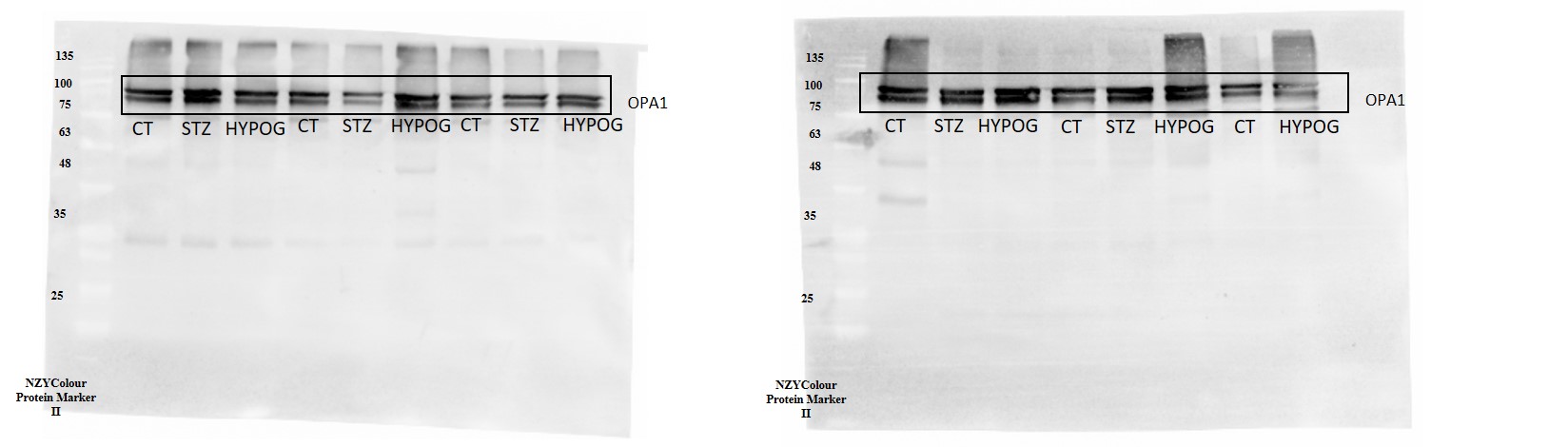

Supplement: Supplementary file 1 [file ijms-22-13470-s001.zip › Figs. Supp/Figure S19.jpg]

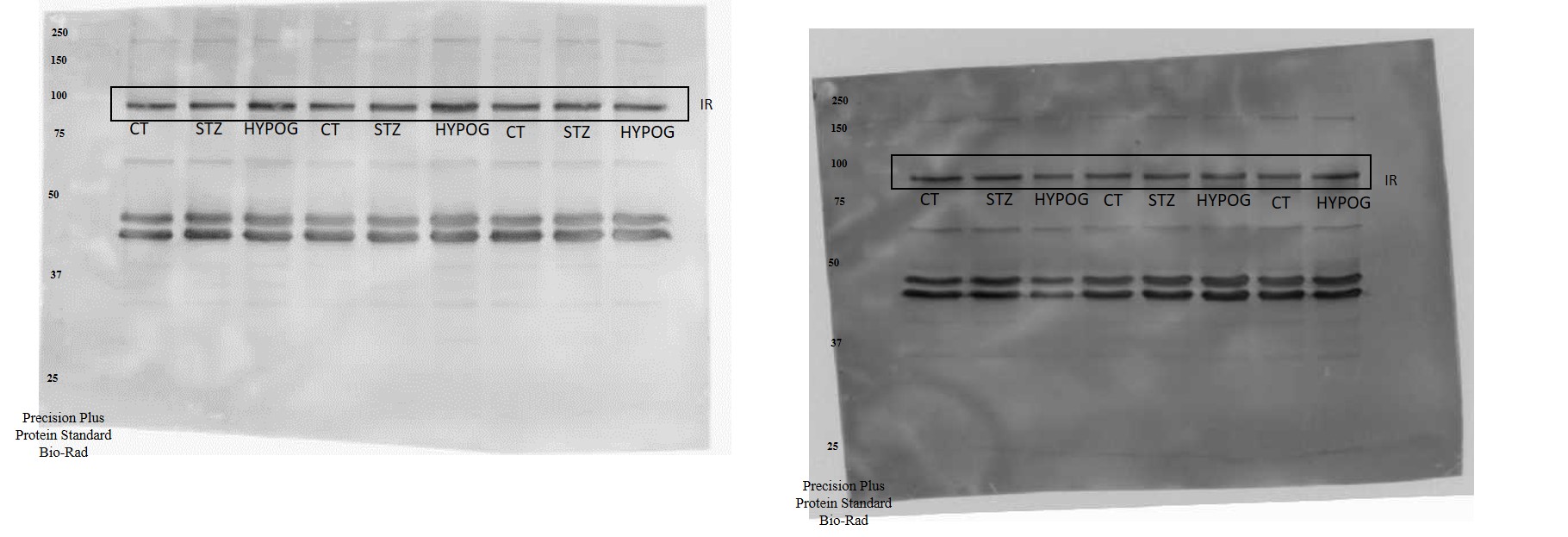

Supplement: Supplementary file 1 [file ijms-22-13470-s001.zip › Figs. Supp/Figure S20.jpg]

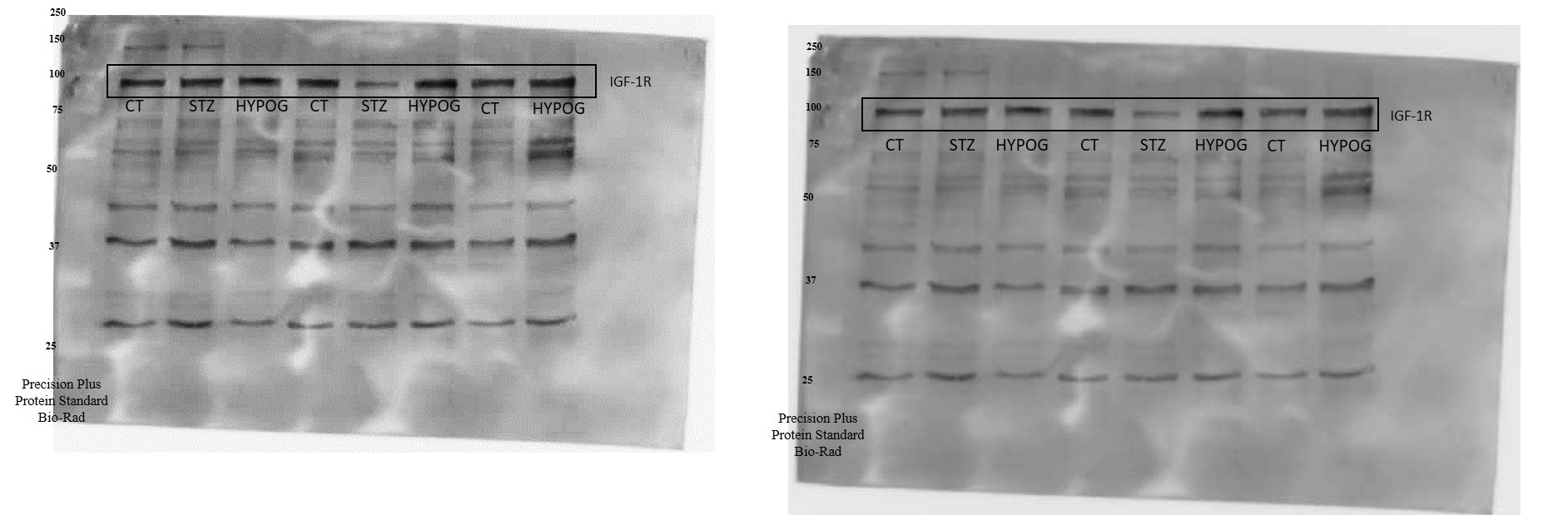

Supplement: Supplementary file 1 [file ijms-22-13470-s001.zip › Figs. Supp/Figure S21.jpg]

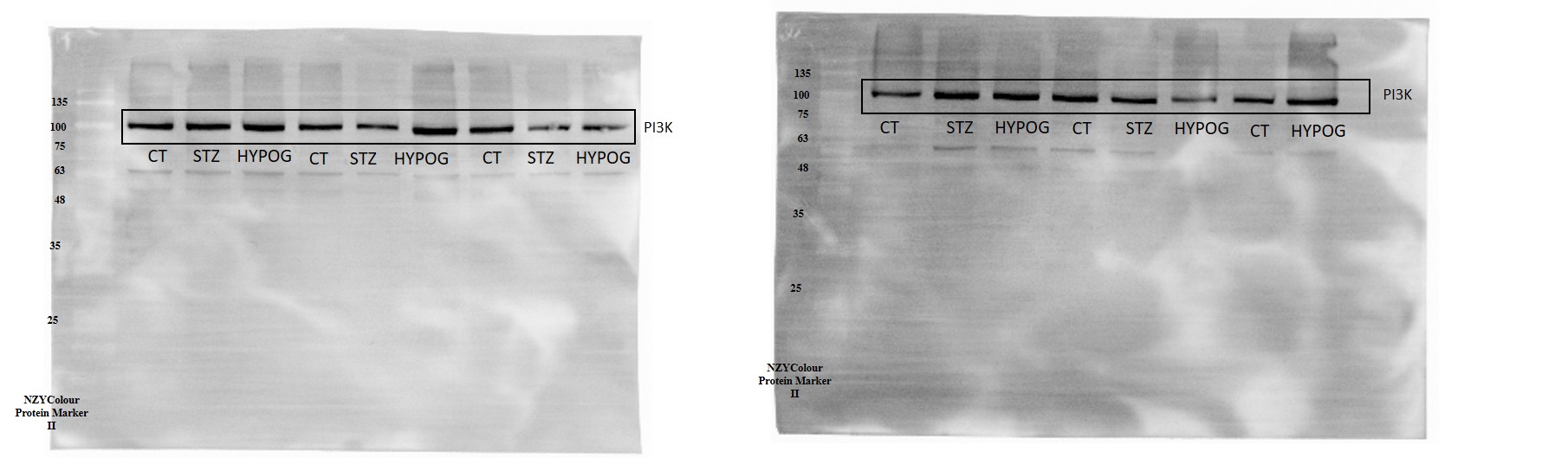

Supplement: Supplementary file 1 [file ijms-22-13470-s001.zip › Figs. Supp/Figure S22.jpg]

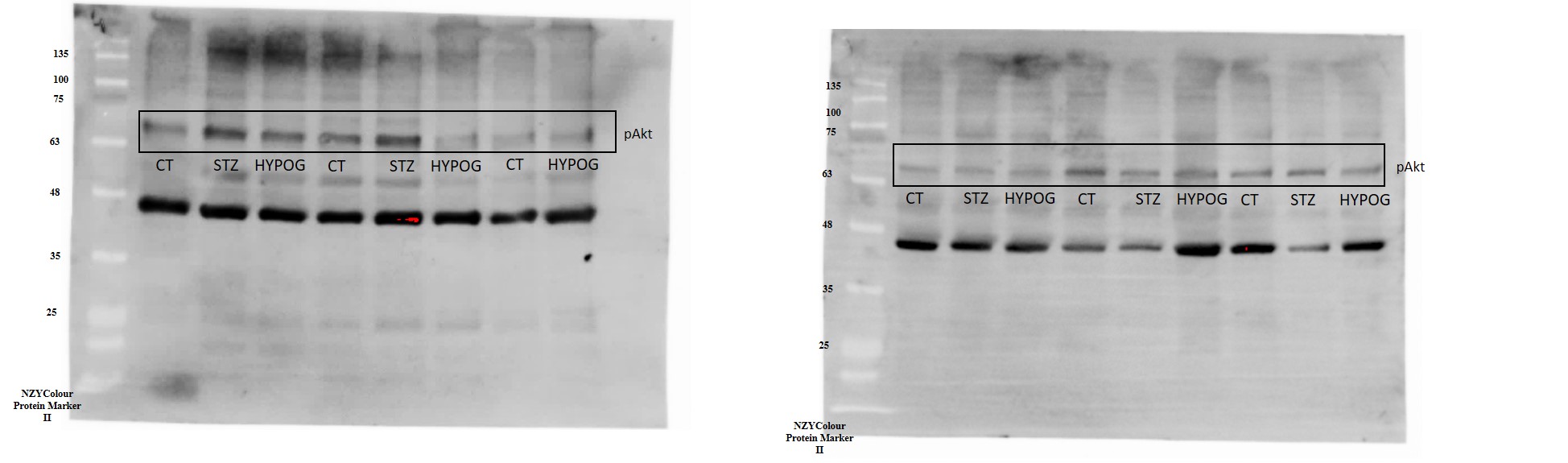

Supplement: Supplementary file 1 [file ijms-22-13470-s001.zip › Figs. Supp/Figure S23.jpg]

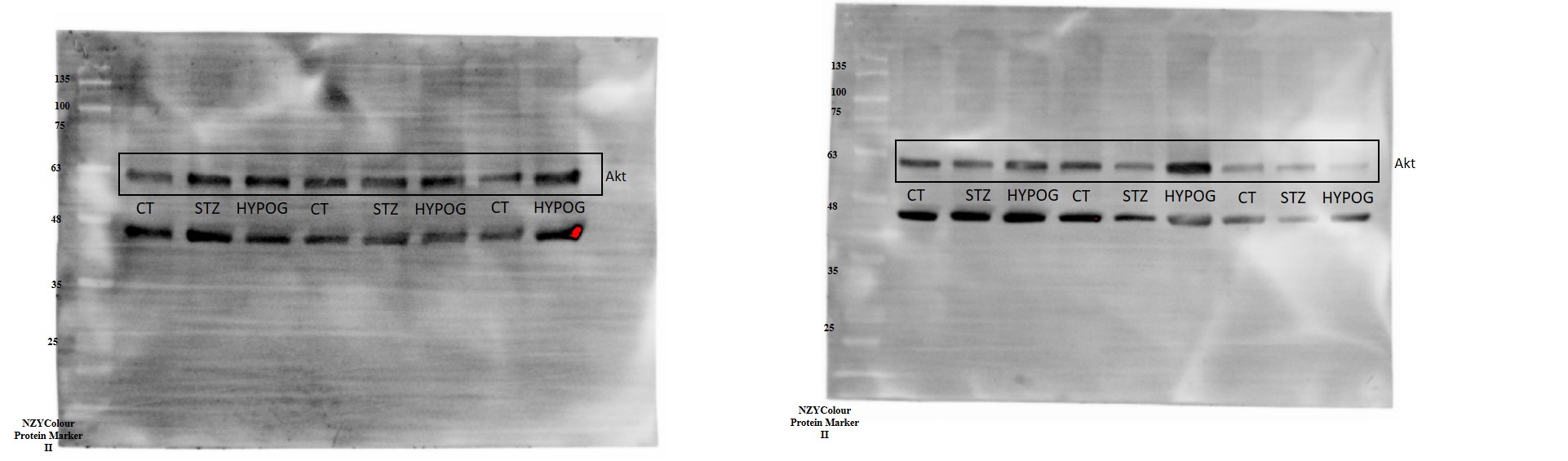

Supplement: Supplementary file 1 [file ijms-22-13470-s001.zip › Figs. Supp/Figure S24.jpg]

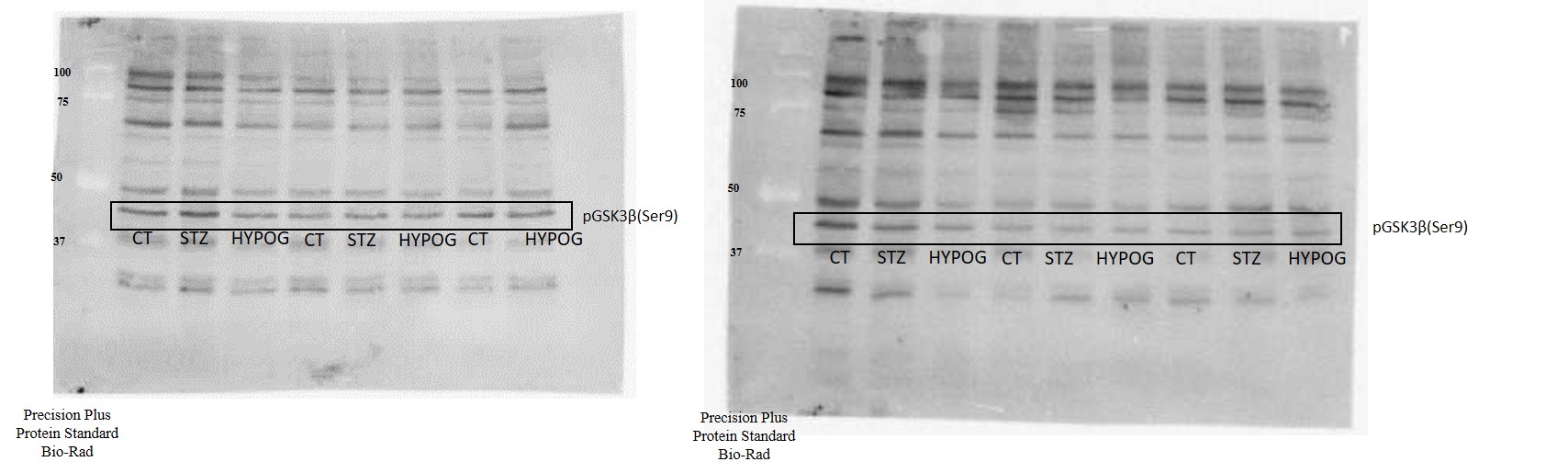

Supplement: Supplementary file 1 [file ijms-22-13470-s001.zip › Figs. Supp/Figure S25.jpg]

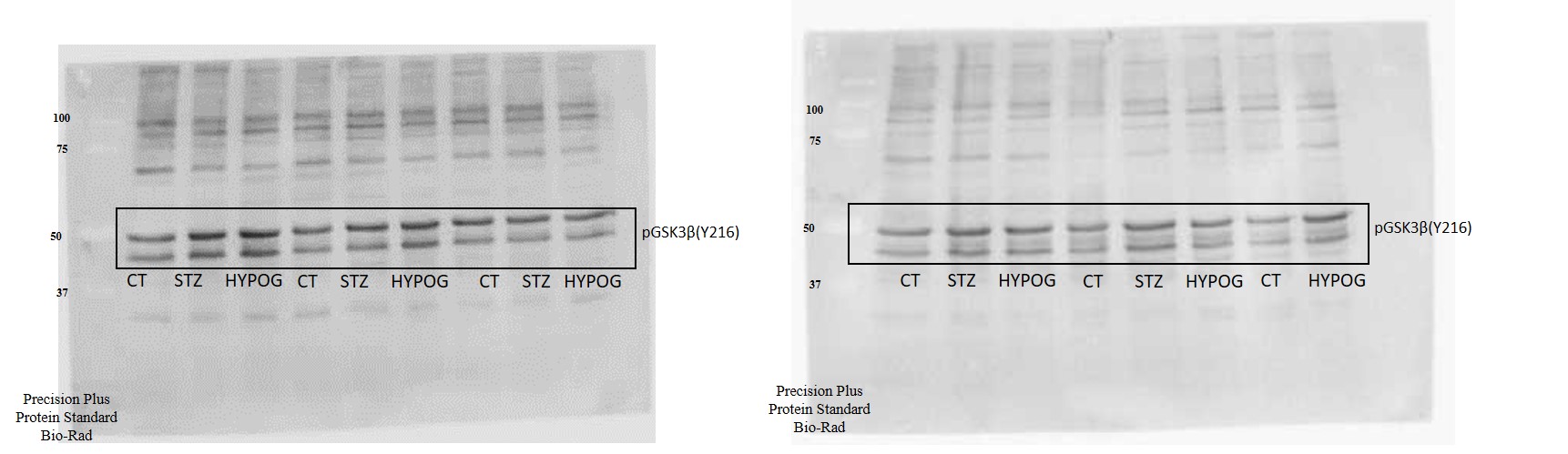

Supplement: Supplementary file 1 [file ijms-22-13470-s001.zip › Figs. Supp/Figure S26.jpg]

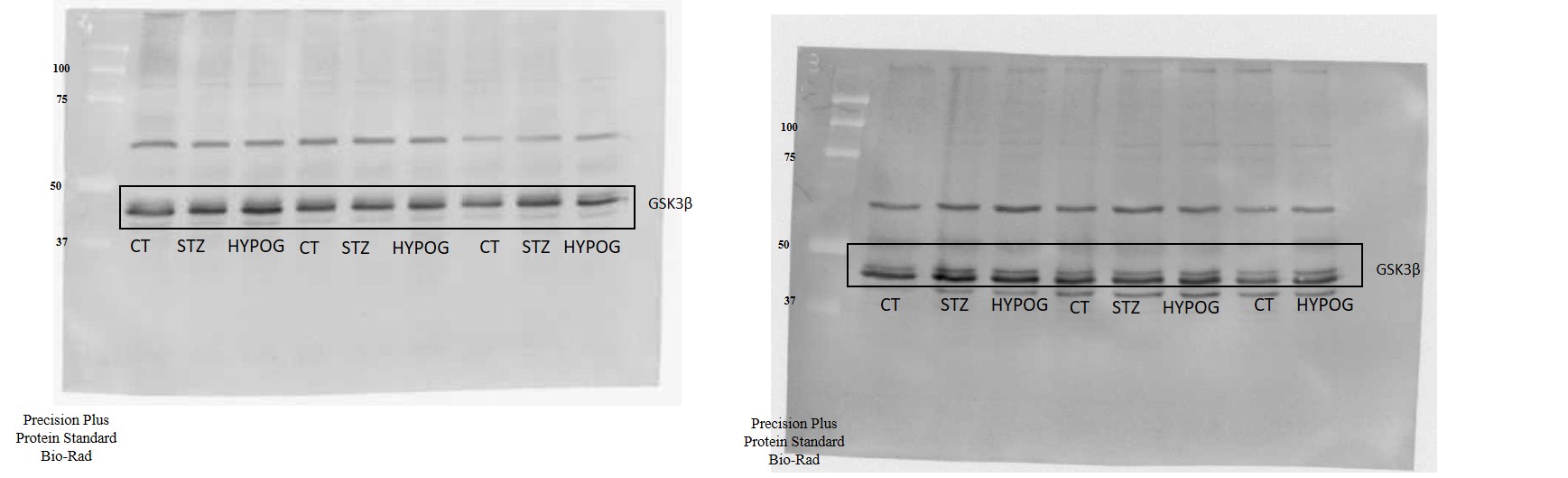

Supplement: Supplementary file 1 [file ijms-22-13470-s001.zip › Figs. Supp/Figure S27.jpg]

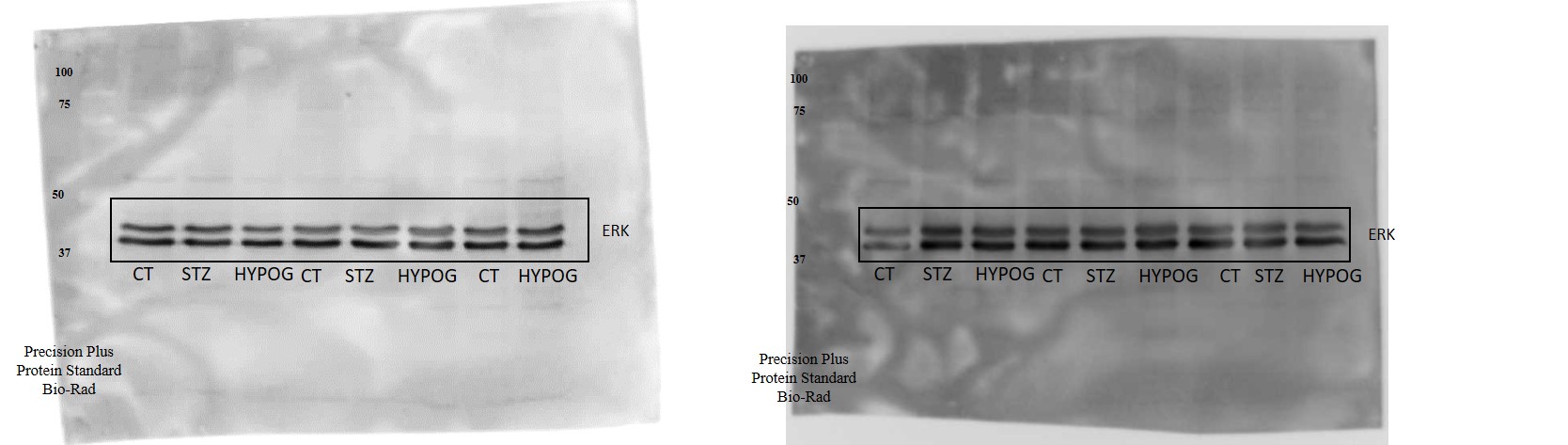

Supplement: Supplementary file 1 [file ijms-22-13470-s001.zip › Figs. Supp/Figure S28.jpg]

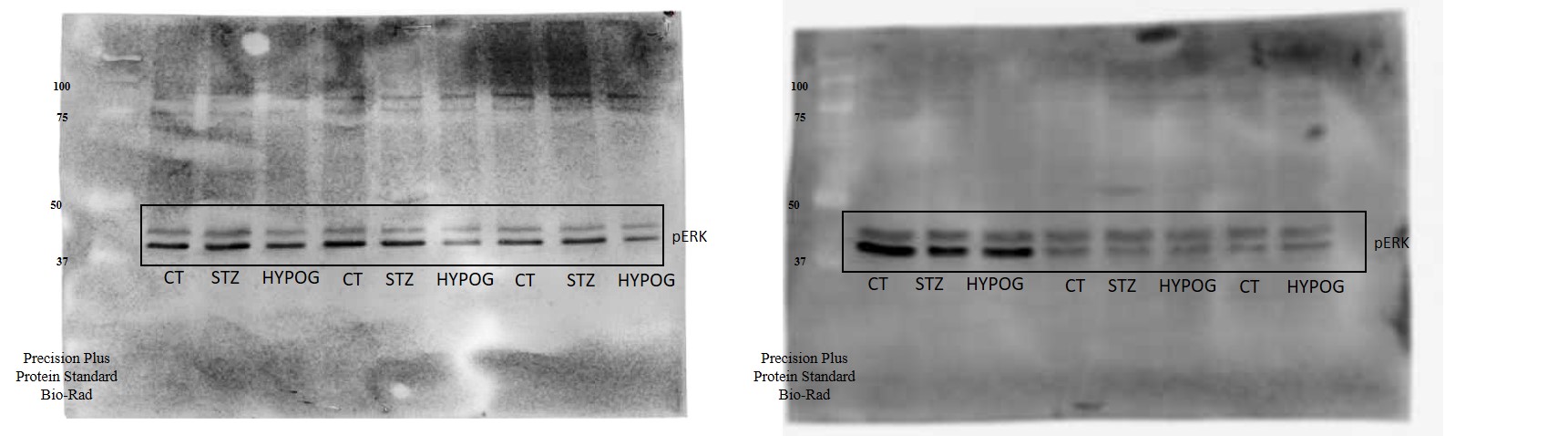

Supplement: Supplementary file 1 [file ijms-22-13470-s001.zip › Figs. Supp/Figure S29.jpg]

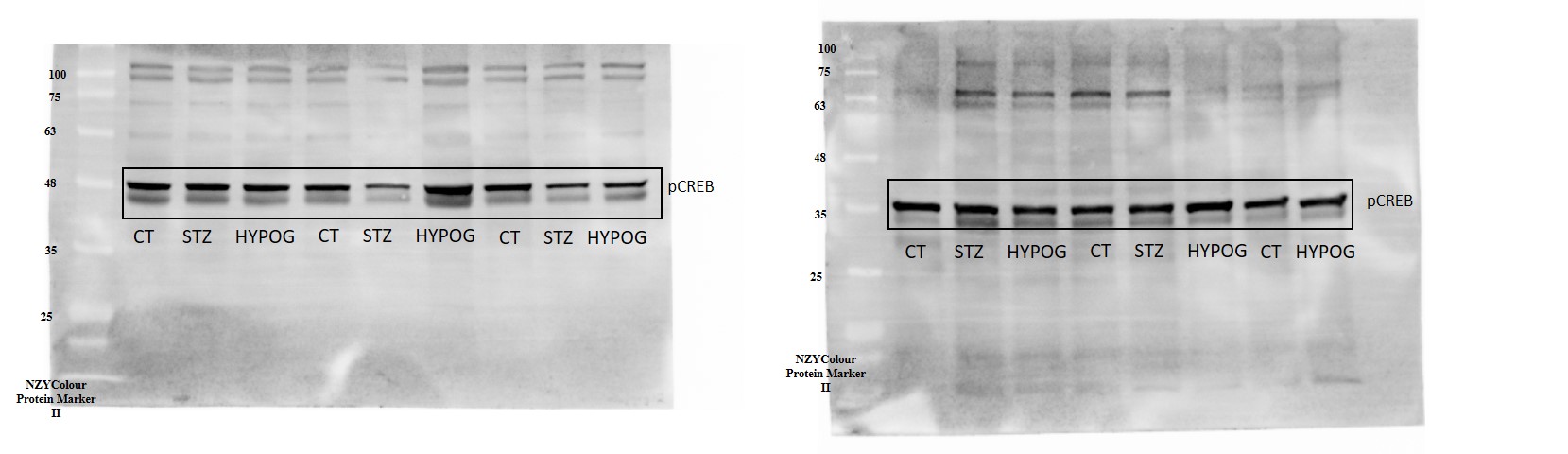

Supplement: Supplementary file 1 [file ijms-22-13470-s001.zip › Figs. Supp/Figure S30.jpg]

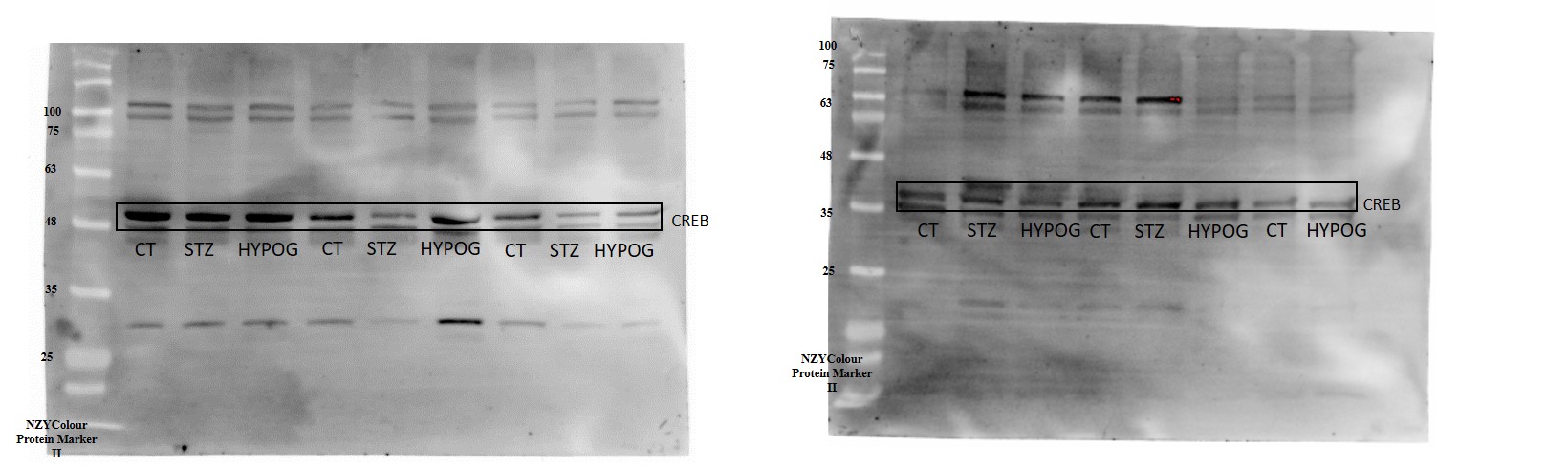

Supplement: Supplementary file 1 [file ijms-22-13470-s001.zip › Figs. Supp/Figure S31.jpg]

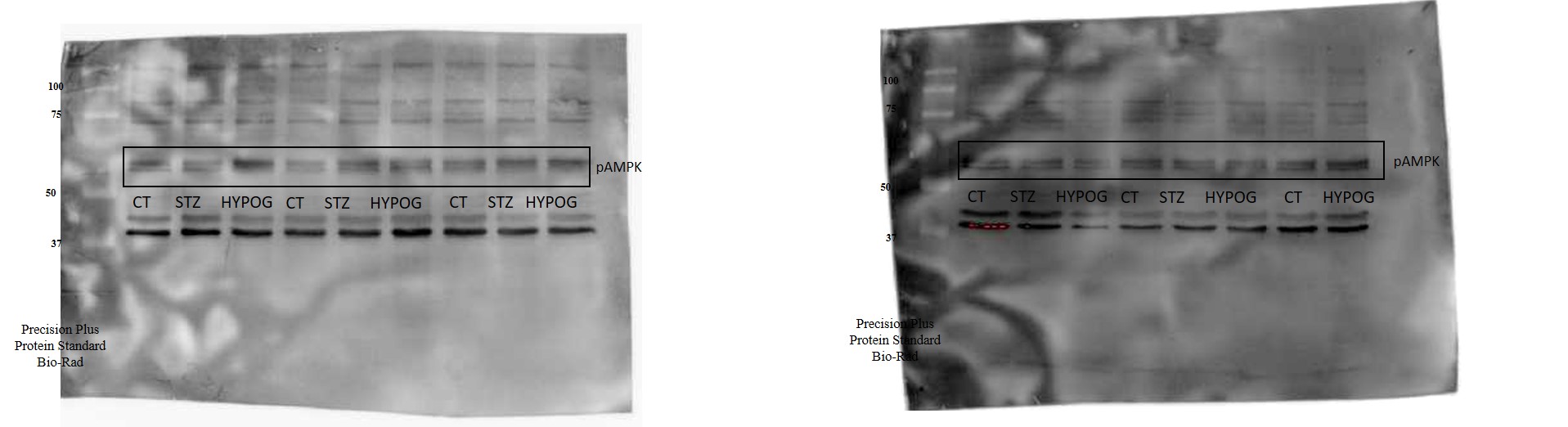

Supplement: Supplementary file 1 [file ijms-22-13470-s001.zip › Figs. Supp/Figure S32.jpg]

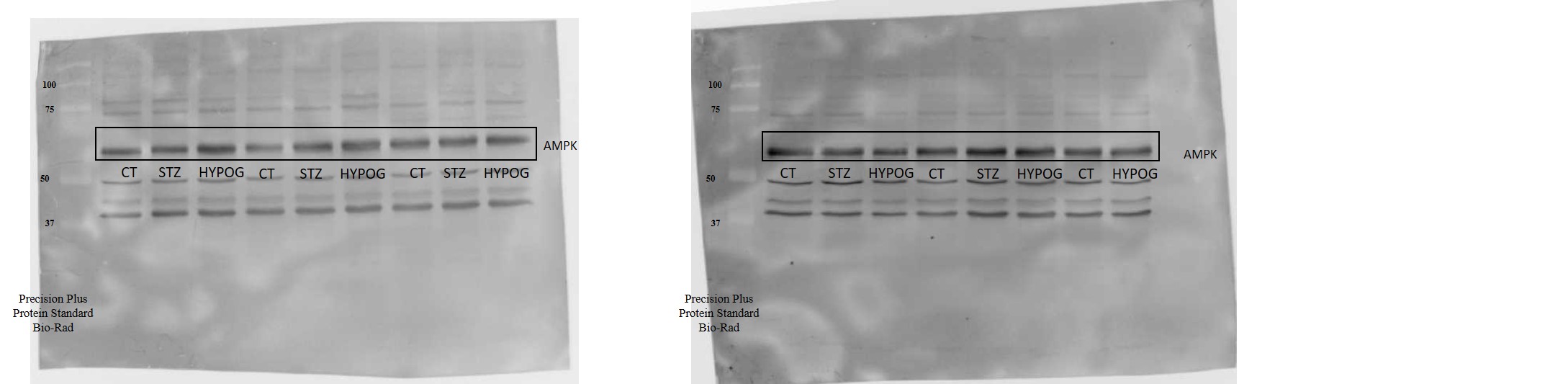

Supplement: Supplementary file 1 [file ijms-22-13470-s001.zip › Figs. Supp/Figure S33.jpg]

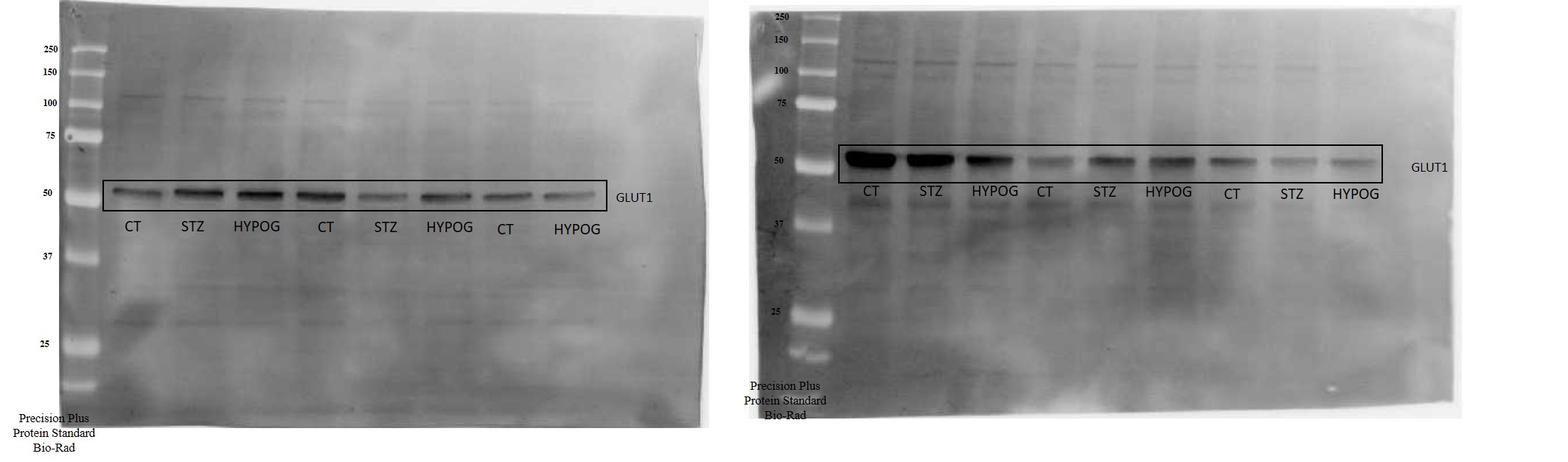

Supplement: Supplementary file 1 [file ijms-22-13470-s001.zip › Figs. Supp/Figure S5.jpg]

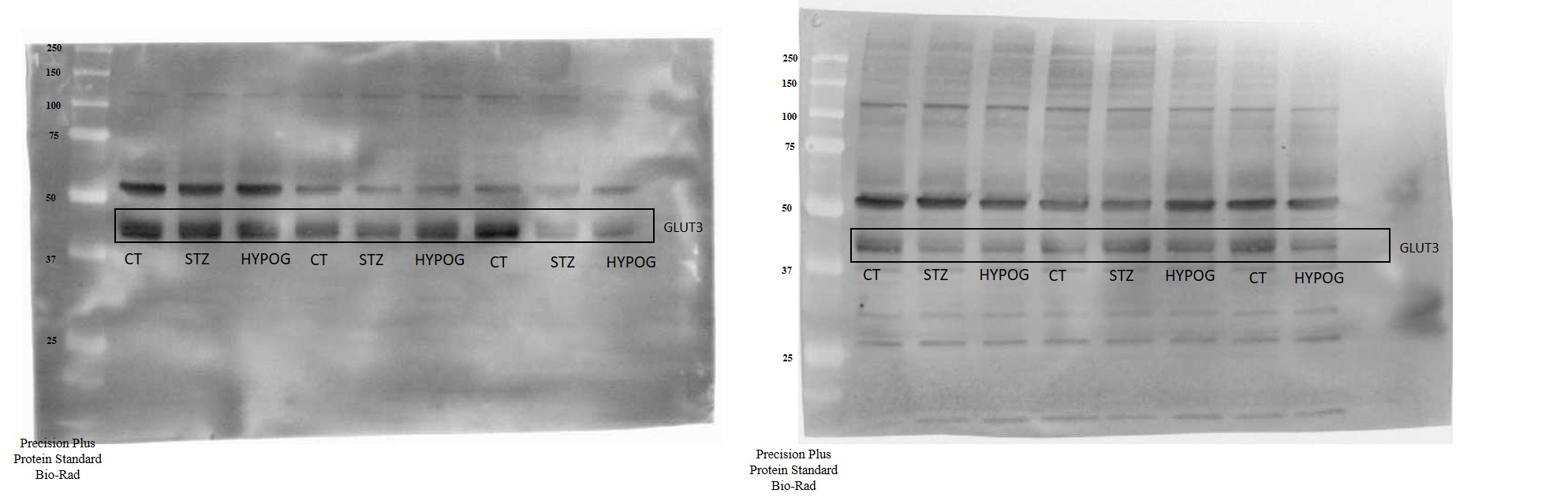

Supplement: Supplementary file 1 [file ijms-22-13470-s001.zip › Figs. Supp/Figure S6.jpg]

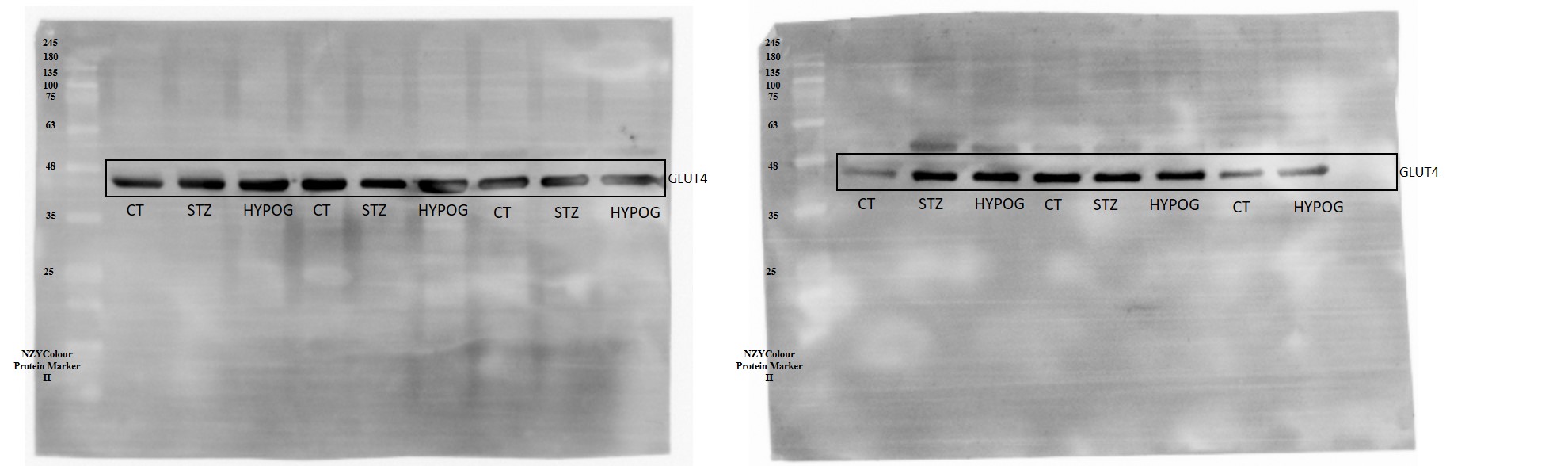

Supplement: Supplementary file 1 [file ijms-22-13470-s001.zip › Figs. Supp/Figure S7.jpg]

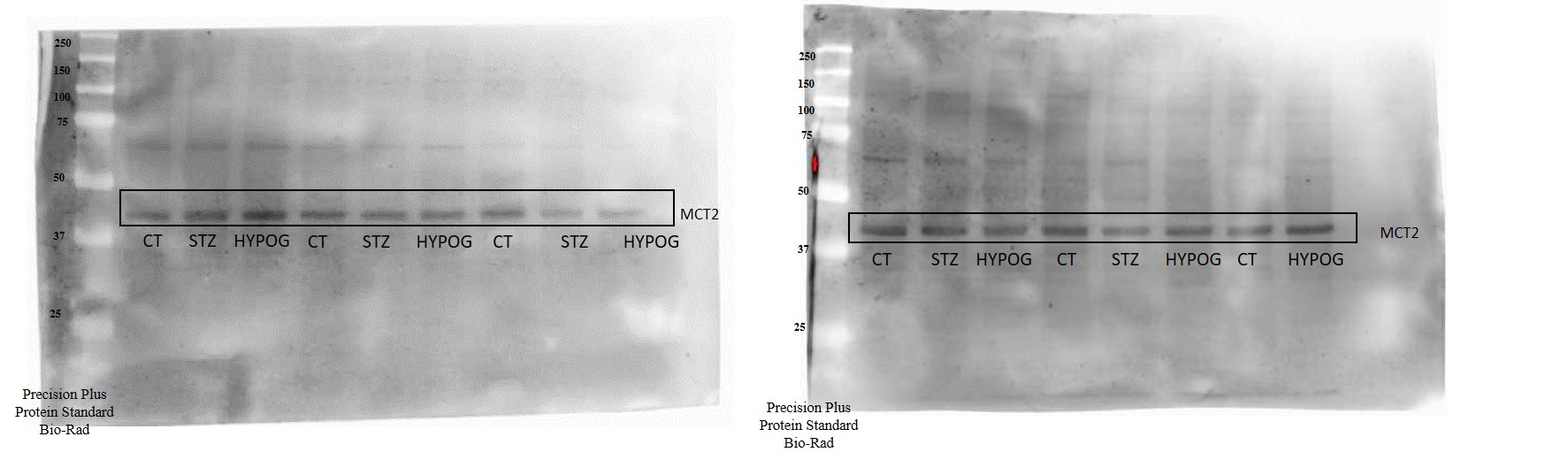

Supplement: Supplementary file 1 [file ijms-22-13470-s001.zip › Figs. Supp/Figure S8.jpg]

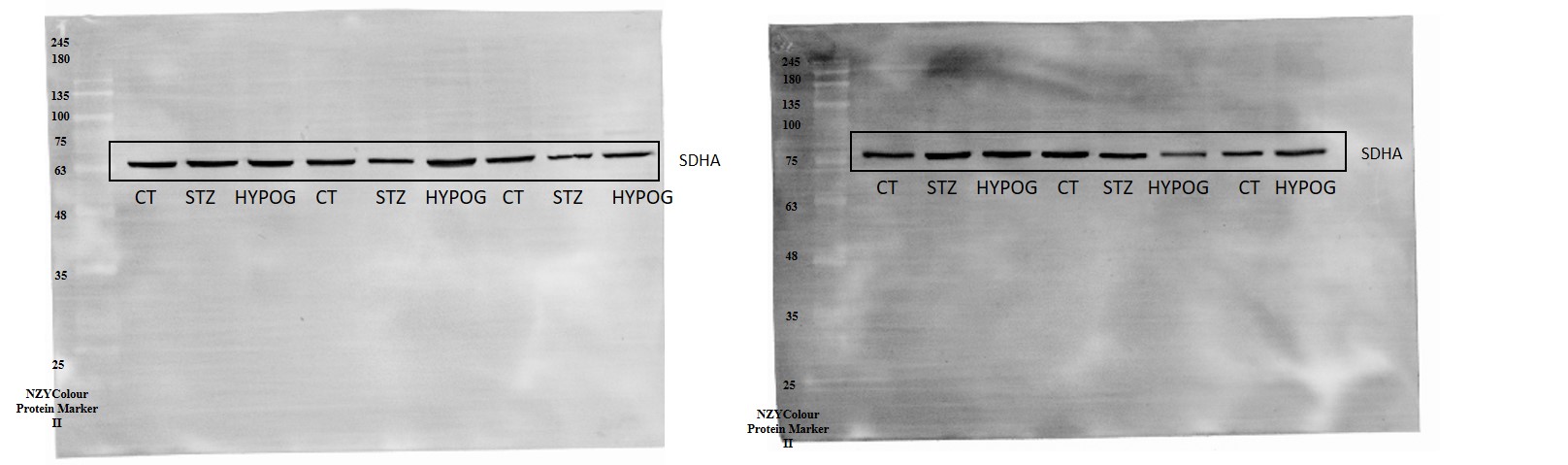

Supplement: Supplementary file 1 [file ijms-22-13470-s001.zip › Figs. Supp/Figure S9.jpg]
